# Cycloartane-Type Saponins, Phytochemical-Rich Extracts, and Sub-Extracts from *Astragalus noeanus* Boiss. Exhibit In Vitro and In Silico Effects on Glucose Metabolism

**DOI:** 10.3390/ph19030352

**Published:** 2026-02-25

**Authors:** Kevser Özdemir-Bayçınar, Timur Hakan Barak, İnci Kurt-Celep, M. Oluş Özbek, Dongdong Wang, Ozan Savaşan, Esra Eroğlu Özkan

**Affiliations:** 1 Department of Pharmacognosy, Faculty of Pharmacy, Fırat University, Merkez, Elazığ 23200, Türkiye; kevser.ozdemir@firat.edu.tr; 2 Institute of Health Sciences, Istanbul University, Fatih, Istanbul 34116, Türkiye; 3 Department of Pharmacognosy, Faculty of Pharmacy, Acıbadem Mehmet Ali Aydınlar University, Atasehir, Istanbul 34755, Türkiye; timur.barak@acibadem.edu.tr; 4 Department of Pharmaceutical Technology, Faculty of Pharmacy, Okan University, Tuzla, Istanbul 34959, Türkiye; inci.celep@okan.edu.tr; 5 Chemical Engineering Department, Gebze Technical University, Gebze, Kocaeli 41400, Türkiye; 6 Molecular Targets Program, Center for Cancer Research, National Cancer Institute, Frederick, MD 21702-1201, USA; dongdong.wang@nih.gov; 7 Department of Pharmaceutical Microbiology, Faculty of Pharmacy, Okan University, Tuzla, Istanbul 34959, Türkiye; ozan.savasan@okan.edu.tr; 8 Department of Pharmacognosy, Faculty of Pharmacy, İstanbul University, Fatih, Istanbul 34116, Türkiye

**Keywords:** *Astragalus noeanus*, antidiabetic activity, α-amylase, DPP IV, PTP1B, AGEs, prebiotic, antioxidant activity, molecular docking

## Abstract

**Background/Objectives**: This study aimed to evaluate the antidiabetic potential of five extracts/sub-extracts and five known cycloartane saponins [astragalosides (AST) I, II, III, IV, and cyclocanthoside E] from *Astragalus noeanus* (AN), using four specific diabetes-related molecular targets. **Methods**: Four diabetes-associated in vitro and in silico targets—protein tyrosine phosphatase 1B (PTP1B), dipeptidyl peptidase IV (DPP IV), α-amylase, and advanced glycation end-products (AGEs)—were employed to obtain comprehensive antidiabetic activity profiles. Additionally, the antioxidant and prebiotic capacities of the extracts/sub-extracts were assessed in vitro. A cycloartane saponin was isolated and structurally characterized. Quantitative analyses of total flavonoids, total saponins, and high-performance thin-layer chromatography (HPTLC) were performed to profile the chemical constituents of the plant material. **Results**: Among the extracts/sub-extracts, the aqueous extract (ANW) exhibited the highest inhibitory effects against all four diabetes-related targets, with inhibition percentages ranging from 83.70% to 93.49%. The methanol extract (ANM) demonstrated significant prebiotic activity comparable to standard controls on two *Lactobacillus* strains. The chloroform extract (ANC) showed the highest flavonoid content and exhibited the strongest antioxidant activity across all assays. ANM yielded the highest saponin content (3250 mg escin equivalent/g). HPTLC quantification revealed that AST IV was the predominant saponin in ANM (14.28 μg/mg) after cyclocanthoside E (117.27 ± 6.71 μg/mg). Among the saponins, AST IV displayed the most potent inhibition in diabetes-related enzyme assays, surpassing reference drugs acarbose and vildagliptin at equivalent concentrations. AST III also demonstrated considerable activity, ranking just below AST IV. Molecular docking studies identified AST II and AST III as the most promising ligands, exhibiting superior binding affinities and stronger hydrogen bonding and hydrophobic interactions with target proteins. Cyclocanthoside E was isolated from *A. noeanus* and evaluated for its antidiabetic effects for the first time, with its structure confirmed by NMR and LC-HRMS analyses. **Conclusions**: This study highlights *Astragalus noeanus* as a promising source for safe and effective antidiabetic agents. The potent activity of the aqueous extract, along with AST IV and AST III, warrants further investigation through clinical trials to validate their therapeutic potential in diabetes management.

## 1. Introduction

*Diabetes mellitus* (DM), the most prevalent disorder of the endocrine system, is characterized by chronic hyperglycemia resulting from defects in insulin secretion, insulin action, or both. When inadequately managed, DM may lead to serious and often irreversible complications such as retinopathy, nephropathy, neuropathy, and atherosclerosis. The multifactorial nature and global epidemic of diabetes have driven the search for more effective, biocompatible, and affordable therapeutic agents [1]. Among these, herbal medicines have drawn increasing interest due to their rich chemical diversity and structural complexity, which often serve as valuable scaffolds for the development of novel antidiabetic drugs [2].

Targeting specific biochemical markers has become a central strategy in diabetes research. Glucagon-like peptide-1 (GLP-1) and glucose-dependent insulinotropic polypeptide (GIP) are key incretin hormones that regulate glucose homeostasis by stimulating insulin secretion. These hormones are rapidly degraded by dipeptidyl peptidase IV (DPP IV), and thus inhibition of this enzyme has become a major therapeutic approach in type 2 diabetes mellitus (T2DM) management [3]. Similarly, protein tyrosine phosphatase 1B (PTP1B) plays a crucial role in insulin signaling, and its inhibition enhances insulin sensitivity [4]. In addition, inhibition of carbohydrate-digesting enzymes such as α-amylase can delay glucose absorption, thereby helping to control postprandial hyperglycemia [5].

In recent years, in vitro and in silico assays have gained prominence as efficient, ethical, and cost-effective tools for investigating biological activities. Enzyme inhibition assays targeting α-amylase, DPP IV, and PTP1B provide high specificity and mechanistic insights [6]. Molecular docking studies, on the other hand, support drug discovery by modeling interactions between bioactive molecules and target proteins [7]. Given that oxidative stress contributes to the onset and progression of diabetes, antioxidants also represent a critical component in preventing related complications [8]. Moreover, the accumulation of advanced glycation end products (AGEs) under hyperglycemic conditions has been strongly implicated in diabetic tissue damage [9]. Another emerging research direction involves the modulation of gut microbiota, which is known to influence glucose metabolism and insulin sensitivity. Notably, polysaccharides from certain medicinal plants may exert prebiotic effects and favorably modulate the gut flora [10,11]. In this context, members of the *Lactobacillus* genus have been shown to ameliorate diabetic symptoms in various studies [12,13].

Medicinal plants have long been recognized as valuable sources of bioactive compounds for the management of chronic diseases, including diabetes mellitus. Numerous phytochemicals demonstrate hypoglycemic effects via multiple pathways, underscoring their key role in diabetes prevention and control. Among these medicinal plants, the genus *Astragalus* (Fabaceae) is one of the largest among flowering plants and is especially well represented in Türkiye, which hosts approximately 450 taxa—about half of which are endemic [14].

*Astragalus noeanus* Boiss. (AN) is one such endemic species, yet no prior studies have investigated its phytochemical composition or pharmacological properties. However, other *Astragalus* species have long been used in traditional medicine to manage diabetes and related conditions [15,16]. In particular, the roots of *A. gummifer*, *A. kurdicus*, *A. brachycalyx*, *A. longifolius*, and *A. halicacabus* have been used for antidiabetic purposes in Eastern Anatolia [17,18,19]. Extensive pharmacological research supports the antidiabetic efficacy of *Astragalus* extracts, primarily attributed to the synergistic action of saponins, polysaccharides, and flavonoids [20]. Among these, astragaloside IV (AST IV) has received considerable attention for its ability to reduce blood glucose levels, improve insulin sensitivity, and protect renal function [21,22]. Cycloastragenol (CAG), the aglycone metabolite of AST IV, and other saponins such as isoastragaloside I, astragaloside II, and formononetin have also demonstrated antidiabetic properties in experimental models [23,24,25]. Furthermore, *Astragalus* polysaccharides have shown β-cell protective effects and improved insulin-related parameters [26,27].

Although the antidiabetic properties of various *Astragalus* species and their key constituents have been previously reported, there remains a significant gap in comparative studies evaluating both in vitro and in silico effects across multiple diabetic targets [28]. Therefore, the present study aimed to fill this gap by investigating the antidiabetic potential of *Astragalus noeanus* root extracts/sub-extracts and its major saponins, providing a preliminary insight into this endemic species. Using chromatographic methods, cyclocanthoside E (CCE)—a cycloartane-type saponin—was isolated, and four astragalosides (AST I–IV) were quantified using high-performance thin-layer chromatography (HPTLC). The antidiabetic activities of the extracts/sub-extracts and pure compounds were evaluated using enzymatic assays targeting α-amylase, PTP1B, DPP IV, and AGEs, along with antioxidant and prebiotic activity tests. Additionally, molecular docking studies were performed to explore the binding interactions between the five *Astragalus* saponins and the aforementioned targets. Total saponin and flavonoid contents were also determined to characterize the chemical composition of the extracts/sub-extracts. This is the first comprehensive study investigating the phytochemical profile and antidiabetic bioactivities of *Astragalus noeanus*, with an integrated approach combining in vitro, in silico, and prebiotic evaluations. The findings aim to contribute to the identification of novel bioactive compounds and provide insights into the mechanisms underlying their therapeutic potential in T2DM.

## 2. Results

### 2.1. Plant Extraction and Isolation

**Figure 1:**
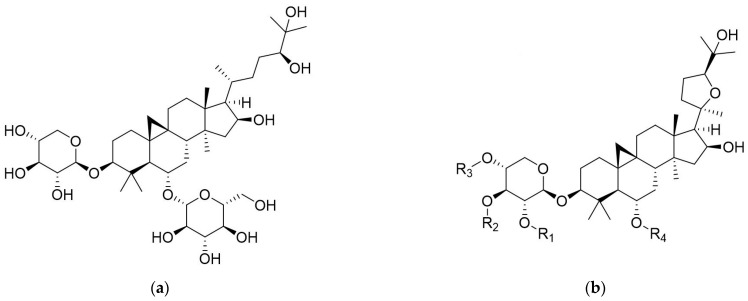
(**a**) Structure of CCE; (**b**) Structures of AST I (R_1_: Ac, R_2_: Ac, R_3_: H, R_4_: Glu), AST II (R_1_: Ac, R_2_: H, R_3_: H, R_4_: Glu), AST III (R_1_: Glu, R_2_: H, R_3_: H, R_4_: H), and AST IV (R_1_: H, R_2_: H, R_3_: H, R_4_: Glu).

Cyclocanthoside E (CCE) was obtained from the butanol sub-extract of the *A. noeanus* plant as a result of various chromatographic studies. CCE was obtained as a white amorphous powder. Its molecular formula of C_41_H_70_O_14_ was determined based on the observed [M−H]^−^ ion peak at *m*/*z* 785.4713 in its negative mode HRESIMS spectrum. The ^1^H NMR spectrum of CCE showed characteristic signals of cycloartane glycoside, including cyclopropane methylene protons and seven methyls in the aglycone moiety, and two sets of *β*-linked sugar units. The ^13^C NMR spectrum contained 41 resonances, 30 of which were attributed to the sapogenol moiety, which were in good agreement with cyclocanthogenin. The full assignments of the ^1^H and ^13^C signals of CCE were achieved utilizing COSY, HSQC, and HMBC experiments (Figures S1–S5). Upon analysis of the NMR data, CCE was determined as 24*S*-cycloartan-3*β*,6*α*,16*β*,24,25-pentaol-3-*O*-*β*-d-xylopyranoside-6-*O*-*β*-d-glucopyranoside, a compound previously isolated from the *Astragalus tragacantha* Habl. (Figure 1a) [29]. It was also isolated from *A. caucasicus*, *A. aureus*, *A. microcephalus*, and several other species previously [30,31,32]. This study marks the first isolation of CCE from *Astragalus noeanus.*

### 2.2. Phytochemical Investigation

The total flavonoid content test results for *A. noeanus* extracts/sub-extracts indicated that the mg quercetin equivalent (mg QE) flavonoid contents were as follows: ANH (11.80 ± 0.11) > ANC (11.72 ± 0.01) > ANM (2.84 ± 0.08) > ANB (2.24 ± 0.03) per 1 g extract, in comparative terms. The flavonoid content in the ANW extract was too low for detection. *Astragalus* roots are well known for their high saponin content. In the current study, the total saponin contents of the extracts/sub-extracts were ranked as follows: ANM (3250 ± 242) > ANW (2056 ± 5.6) > ANB (1998 ± 96) > ANC (1732 ± 23) > ANH (862 ± 21), expressed in mg escin equivalents per g of extract.

**Figure 2:**
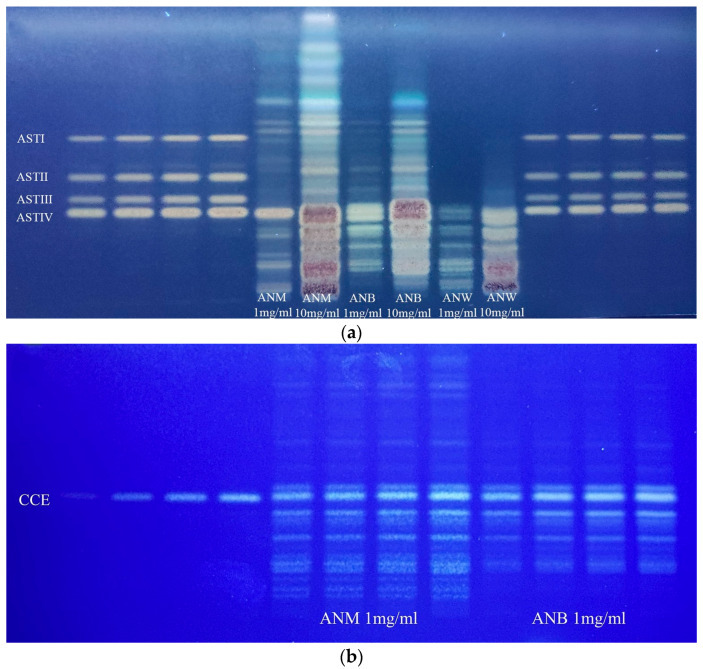
(**a**) HPTLC chromatograms of ASTs I, II, III, and IV. ASTs (100 µg/mL) were applied to the plate on both sides of the extracts/sub-extracts in increasing volumes (1, 2, 4, 8 µL). The extracts were applied in volumes of 20 and 30 µL, respectively. Mobile phase: CHCl_3_/MeOH/EtOAc/H_2_O (13:7:2:2) (*v*/*v*/*v*/*v*); Derivatization: 10% sulfuric acid in EtOH. Visualization: 366 nm. (**b**) HPTLC chromatogram of CCE. CCE (100 µg/mL) was applied to the left side of the extracts/sub-extracts in increasing volumes (1, 2, 4, 8 µL). Mobile phase: CHCl_3_/MeOH/H_2_O (11:5:1) (*v*/*v*/*v*); Derivatization: 10% sulfuric acid in EtOH. Visualization: 366 nm.

This study assessed five *Astragalus* saponins, including AST IV (Figure 1b). Quantification of these saponins in AN was conducted by using HPTLC (Table 1, Figure 2). The maximum concentration of AST IV was identified in the ANB extract (31.25 μg/mg). The AST IV content of the total methanol extract was 14.28 μg/mg. The AST II component was quantified as 3.5 μg/mg in the butanol sub-extract of AN. The contents of ASTs I and II in other extracts were measured in trace quantities. The CCE content of the AN was quantified as 117.27 μg/mg in ANM and 197.13 μg/mg in ANB.

### 2.3. In Vitro Diabetes-Related Bioactivity Assays of A. noeanus Extracts/Sub-Extracts and Saponins

**Figure 3:**
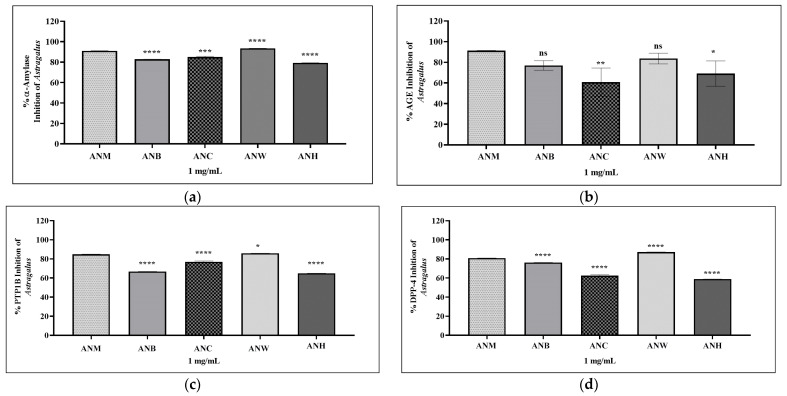
Graphics for diabetes-related enzyme inhibition results of *A. noeanus* extracts and sub-extracts. Statistical analysis was performed using one-way ANOVA followed by Dunnett’s multiple-comparison test. *p* ≤ 0.05 (*), *p* ≤ 0.01 (**), *p* ≤ 0.001 (***), *p* ≤ 0.0001 (****), ns = not significant (*p* > 0.05). Each analysis included the standard reference inhibitor at different concentrations (31.25–1000 µg/mL). Bars represent mean ± SD (*n* = 3). ANM: *A. noeanus* methanol extract; ANB: *A. noeanus* butanol sub-extract; ANC: *A. noeanus* chloroform sub-extract; ANW: *A. noeanus* water sub-extract; ANH: *A. noeanus* hexane extract. (**a**) α-Amylase inhibition graph of *A. noeanus*; (**b**) AGEs inhibition graph of A. noeanus; (**c**) PTP1B inhibition graph of *A. noeanus*; (**d**) DPP IV inhibition graph of *A. noeanus*.

**Figure 4:**
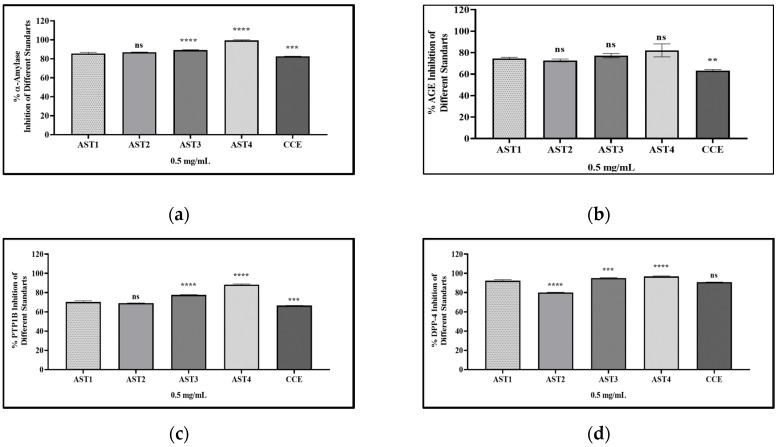
Graphics for diabetes-related enzyme inhibition results of *Astragalus* saponins. Statistical analysis was performed using one-way ANOVA followed by Dunnett’s multiple-comparison test. *p*≤ 0.01 (**), *p* ≤ 0.001 (***), *p* ≤ 0.0001 (****), ns = not significant (*p* > 0.05). Each analysis included the standard reference inhibitor at different concentrations (31.25–1000 µg/mL). Bars represent mean ± SD (*n* = 3). AST I: astragaloside I; AST II: astragaloside II; AST III: astragaloside III; AST IV: astragaloside IV; CCE: cyclocanthoside E. (**a**) α-Amylase inhibition graph of the saponins; (**b**) AGEs inhibition graph of the saponins; (**c**) PTP1B inhibition graph of the saponins; (**d**) DPP IV inhibition graph of the saponins.

The in vitro inhibitory actions against α-amylase, PTP1B, DPP IV, and AGEs associated with AN were displayed in Table 2 and Figure 3. Furthermore, activities of the ASTs I-IV and CCE were presented in Table 3 and Figure 4. Full inhibition data for standard inhibitors at all concentrations are in Table S1. The results indicated that the percent α-amylase inhibition values of the extracts/sub-extracts were ranked as follows: ANW > ANM > ANC > ANB > ANH. The most potent extracts/sub-extracts in the α-amylase inhibitory activity assay were the water sub-extract and the methanol extract. ANM (93.49%) exhibited more inhibition than 1000 μg/mL of acarbose (92.40%). The lowest activity was observed in ANH (79.31%). All extracts/sub-extracts showed significant α-amylase inhibition activity.

All extracts/sub-extracts were evaluated for their inhibitory effects on PTP1B. ANW showed the highest inhibition compared to the others. The inhibitory potential of the extracts/sub-extracts on PTP1B was sorted as follows: ANW > ANM > ANC > ANB > ANH. The present work assessed ASTs I-IV and CCE for their potential to inhibit PTP1B, revealing that ASTs IV and III had the highest inhibition rates (88.37% and 77.58%, respectively), and they showed significantly better inhibitory activity compared to AST I, AST II, and CCE.

This research provided initial insights into the DPP IV inhibition of the extracts/sub-extracts and saponins. ANW had the highest DPP IV inhibitory activity at 87.26%, while the hexane extract demonstrated the lowest activity at 58.85%. Among the saponins, the DPP IV inhibitory activities of AST III (95.21%) and AST IV (96.77%) were higher than the inhibitory activity of vildagliptin (93.21%), at the same concentration of 0.5 mg/mL.

Among the extracts, ANM exhibited the highest AGEs inhibitory activity with 91.38%, while ANC had the lowest activity with 60.83%. 1 mg/mL of ANM exhibited a higher anti-glycation activity compared to 500 μg of quercetin, which demonstrated 85.44% activity. ASTs III and IV exhibited the highest activity, with inhibitions exceeding 77%, in the AGEs inhibition experiment.

The in vitro prebiotic activities of the extracts/sub-extracts on specific *Lactobacillus* strains were assessed. The growing stimulation effects of the extracts/sub-extracts on the probiotics were shown in Table 4.

ANM demonstrated the highest increase in probiotic biomass for GG and LP strains. Its activity on GG growth was comparable to the standards, whereas it was significantly higher than the standards for the LP strain. No significant activity was observed in *Lactobacillus reuteri* after treatment.

### 2.4. Antioxidant Activity

The results of the TOAC, FRAP, CUPRAC, and DPPH antioxidant activity assays of AN extracts/sub-extracts are presented in Table 5. Among all the extracts, ANC exhibited the strongest antioxidant activity across all assays (TOAC: 107.71 mg AAE; FRAP: 0.35 mM FeE; CUPRAC: 65.61 mg AAE, DPPH: 44.34 mg BHTE per gram of extract). Furthermore, ANH showed the lowest activity in all experiments.

### 2.5. Molecular Docking Results

**Figure 5:**
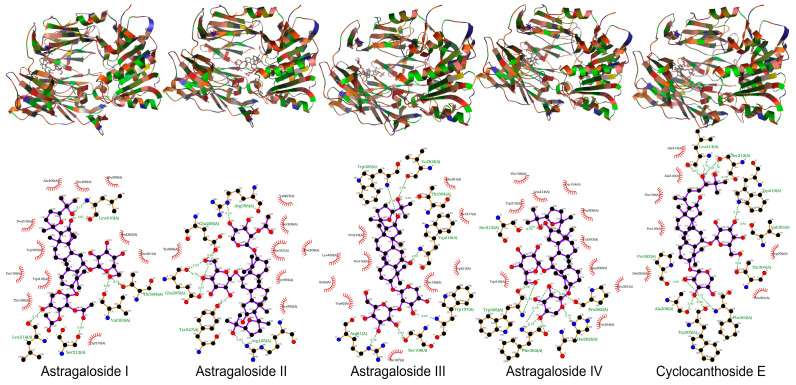
The binding geometries of molecules astragalosides I–IV and cyclocanthoside E in 1J2E, as computed by Autodock-Vina. The top and the bottom figures represent the 3D binding geometries and 2D interactions, respectively.

**Figure 6:**
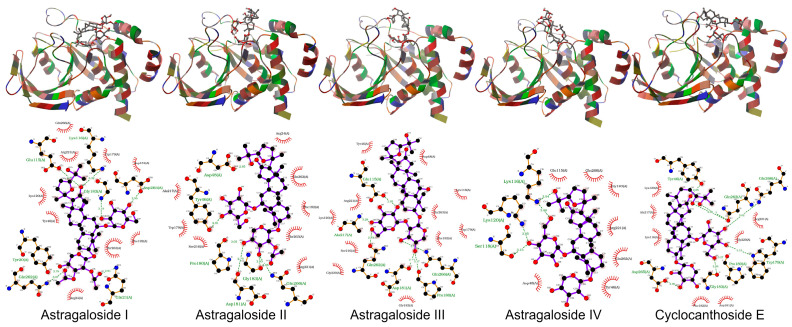
The binding geometries of molecules astragalosides I–IV and cyclocanthoside E in 1NNY, as computed by Autodock-Vina. The top and the bottom figures represent the 3D binding geometries and 2D interactions, respectively.

**Figure 7:**
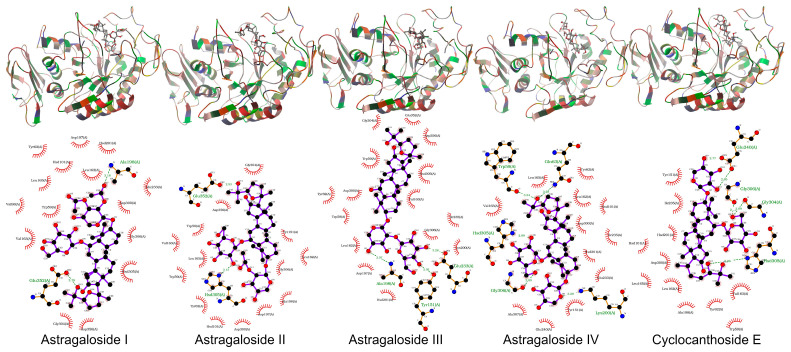
The binding geometries of molecules astragalosides I–IV and cyclocanthoside E in 1OSE, as computed by Autodock-Vina. The top and the bottom figures represent the 3D binding geometries and 2D interactions, respectively.

**Figure 8:**
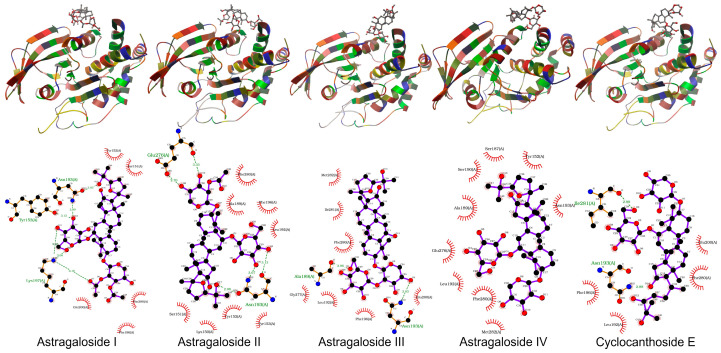
The binding geometries of molecules astragalosides I–IV and cyclocanthoside E in 1T49, as computed by Autodock-Vina. The top and the bottom figures represent the 3D binding geometries and 2D interactions, respectively.

**Figure 9:**
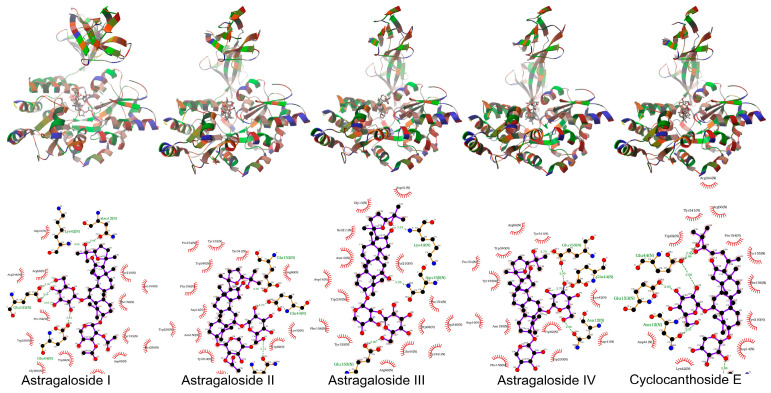
The binding geometries of molecules astragalosides I–IV and cyclocanthoside E in 3O3U, as computed by Autodock-Vina. The top and the bottom figures represent the 3D binding geometries and 2D interactions, respectively. Docking scores (kcal/mole) of the ligands astragaloside I (AST I), astragaloside II (AST II), astragaloside III (ASTIII), astragaloside IV (ASTIV), and cyclocanthoside E (CCE) on the given proteins as computed by Autodock and Autodock-Vina. The calculated inhibition constants (Ki) are given in micromolar (μM) or millimolar (mM).

In this study, both AutoDock 4.2.6 and AutoDock Vina 1.1.2 produced consistent results in docking studies of the protein-ligand complexes. The 3D geometries of the docked ligands at their lowest energy conformations, alongside 2D interaction maps generated by AutoDock Vina, which detail the key interactions within the protein active sites, were presented in Figure 5, Figure 6, Figure 7, Figure 8 and Figure 9. For a more comprehensive view, the complete set of interaction maps, including those from the blind docking simulations, was provided in Figures S6–S12.

Table 6 summarizes the docking scores and inhibition constants (calculated via AutoDock) for each protein-ligand pair at their respective active sites. To identify the most promising ligands based on docking performance, the docking scores were summed row-wise for each ligand-protein pair.

The selectivity of ligands for their target proteins is key to their therapeutic efficacy. Docking studies showed that AST II and AST III selectively bound to the active sites of their targets. As shown in Figure 5, Figure 6, Figure 7, Figure 8 and Figure 9 and Table 7, the 2D interaction maps revealed the key hydrogen bonding (H-bond) and hydrophobic interactions that stabilize the protein-ligand complexes.

ASTs II and III showed the highest docking scores, strong hydrogen bonding, and hydrophobic interactions with target proteins, while CCE exhibited the lowest potential.

## 3. Discussion

Plants provide a rich array of bioactive compounds with varying pharmacological properties, which are employed in the pharmaceutical industry extensively. Saponins, flavonoids, and polysaccharides constitute the predominant bioactive constituents of the *Astragalus* genus. To determine the abundance of specific *Astragalus* saponins in *A. noeanus* and their effects on diabetes-related targets, the CCE, which was isolated from the plant, and four ASTs were evaluated. Quantification of the mentioned saponins in AN extracts/sub-extracts was conducted by using HPTLC. AST IV is a key saponin from *Astragalus* species and is employed as a reference compound in the standardization of the plant.

Previous studies have reported varying levels of astragalosides in different *Astragalus* species and root parts. A study has reported varying levels of astragalosides in commercial *Astragalus memranaceus* 50% hydroalcoholic root extracts. In the samples, AST I content was reported between 0.0003 and 0.24 mg/g, and AST IV between 0.011 and 0.84 mg/g, as determined by ultra-performance liquid chromatography (ESI-QTrap-MS/MS) [33]. In *Astragalus membranaceus* var. *monghulicus* methanol extract, the average AST IV content was reported as 0.16 mg/g [34]. Another study investigating Radix Astragali from different regions showed that astragalosides were significantly more concentrated in thin roots compared to thick roots, and their distribution varied between xylem and bark: AST I ranged from 0.027 to 3.295 mg/g, AST II from 0.012 to 0.493 mg/g, and AST IV from 0.012 to 0.376 mg/g in the methanolic extracts [35]. These data are not directly comparable due to differences in species, the analyzed plant part, and extraction methods; nevertheless, they collectively indicate that astragaloside content is highly variable across species and depends on the methods used. In addition, there is limited data on AST III and CCE concentrations in *Astragalus* species.

The study represents the first application of HPTLC for detecting the CCE compound, as well as the first measurement of saponins from *A. noeanus*. The highest concentration of AST IV was found in the ANB extract (31.25 μg/mg). Despite differences in extraction methods and species, the AST IV content was found to be relatively higher compared to previous studies [33,34,35,36]. AST II was only quantified in the ANB as 3.5 μg/mg. ASTs I and II were present in trace amounts in the other extracts. The elevated AST IV levels and the trace of low quantities of ASTs I and II in the extracts can be explained by the conversion of these compounds into AST IV during extraction. The CCE content of the AN was the highest among saponins and quantified as 117.27 μg/mg in the total extract. *A. noeanus* constitutes an important source of CCE and ASTs. Saponins were either undetectable or detected only in trace amounts in the lipophilic hexane and chloroform extracts/sub-extracts.

Phenolic compounds have shown potential in managing chronic degenerative disorders, including diabetes and other metabolic diseases [37]. Astragalin, kaempferol, apigenin, rutin, formononetin, calycosin, and ononin are the most common flavonoids found in *Astragalus* species. Moreover, formononetin administration in diabetic rats significantly lowered blood glucose levels and improved glucose tolerance, insulin sensitivity, and lipid profile [23]. The flavonoid content in *A. noeanus* extracts/sub-extracts was highest in the non-polar extracts, with ANH exhibiting the highest concentration. Furthermore, the concentration of flavonoids in the ANW extract was insufficient for detection. *Astragalus* roots are known for their high saponin content, with the highest levels found in the ANM extract. Other polar sub-extracts, ANW and ANB, were also found to have high saponin content, respectively. The significant activity observed in the total extract may be attributed to the combined effects of the tested active constituents, particularly flavonoids and saponins, while other phytochemical groups, including polysaccharides reported by earlier studies [27], might also contribute to the overall effect.

A variety of in vitro assays were conducted to evaluate the antidiabetic properties of the extracts/sub-extracts and saponins. The highest *α*-amylase inhibition was observed in 1 mg/mL ANW and ANM, where ANM exceeded the inhibition of 1 mg/mL acarbose. The lowest activity was observed in ANH, and all extracts/sub-extracts showed significant inhibition. Several members of the genus *Astragalus* have also been identified as α-amylase inhibitors. α-amylase inhibitory activity was measured in total extracts of *A. bruguieri* (0.51 mmol ACAE/g), *A. hirsutus* (0.80 mmol ACAE/g), and *A. ponticus* (0.60 mmol ACAE/g). Furthermore, in these investigations, the roots exhibited comparable inhibitions to those shown in other plant parts, including flowers, stems, and leaves [38,39,40]. Considering that previous studies have reported higher flavonoid concentrations in the aerial parts and greater amounts of saponins and polysaccharides in the roots, it may be concluded that α-amylase inhibition originates from multiple molecular groups. Consistent with the related studies, α-amylase inhibition was observed to be relatively high in the total methanol extract.

The overexpression of PTP1B and the imbalance between kinases and phosphatases lead to insulin resistance. Consequently, the inhibition of PTP1B is a key target in the management of T2DM [4]. All extracts/sub-extracts were tested for PTP1B inhibition, revealing that each extract exhibited differing levels of inhibition. The ANW exhibited the best results as compared to other extracts based on their percentage inhibitions. The PTP1B inhibitory potential of *Astragalus* extracts and polysaccharides, demonstrated in previous in vitro and in vivo studies, was in line with the findings for AN in this study [41,42]. Among the compounds, ASTs IV and III showed the most significant inhibitory effects. Additionally, at a concentration of 500 mg/mL, the reference compound ursolic acid exhibited 90.75% inhibition, whereas AST IV demonstrated an enzyme inhibition that was merely 2.38% lower. AST IV has recently gained attention for its potential in preventing and treating diabetes and associated complications due to its ability to lower blood glucose levels, reduce insulin resistance, and protect renal function [28]. In a former in vitro study, AST IV selectively inhibited PTP1B and showed an IC_50_ of 10.34 ± 0.54 μM. The Western blot assay in the same study revealed suppression following AST IV treatment, and in silico analysis confirmed this conclusion [43].

Although research suggested that AST II and certain *Astragalus* extracts/sub-extracts enhance insulin sensitivity and activate GLP-1, there was inadequate data to evaluate the DPP IV inhibitory potential of *Astragalus* and its primary constituent [44,45,46,47]. This study offered preliminary data on the DPP IV inhibitory activity of AN and key *Astragalus* saponins. The DPP IV inhibitory activities of ASTs III-IV were higher than the inhibitory activity of vildagliptin, at an equivalent concentration of 0.5 mg/mL. Furthermore, ANW had the highest DPP IV inhibitory activity within the extracts/sub-extracts. When evaluating diabetes-related test data, the inhibitory potential of ASTs III and IV and the total AN extract on DPP IV and α-amylase was particularly significant.

At a concentration of 1 mg/mL, ANM showed slightly lower anti-glycation activity (2.8% less) than 1 mg/mL quercetin, while exhibiting a higher inhibitory effect compared to 500 µg/mL quercetin. Like other tests, ASTs III and IV showed the best activity among pure substances in the AGEs inhibition experiment. Our findings corroborated previous research indicating that *Astragalus* extracts/sub-extracts and astragalosides inhibit the production of AGEs [40,48]. Our previous research showed that *A. kurdicus* total methanol extract inhibited AGEs with an 85.63% inhibition [46]. The findings in this study, in conjunction with the literature, suggest that ASTs IV and III may be suitable for managing diabetes and associated complications. Further research is needed on safe human dose limits, side effects, and clinical outcomes [28].

The gut microbiota is implicated in obesity, non-alcoholic fatty liver disease, insulin resistance, and chronic inflammation, all of which are associated with the onset of T2DM. Clinical studies indicated that HbA1c and serum cholesterol levels in T2DM patients were lowered following *Lactobacillus reuteri* treatment. Likewise, certain investigations demonstrated that the metabolic effects were linked to *Lactobacillus rhamnosus* GG and *Lactobacillus paracasei* strain [11,49]. To evaluate the possible contributions to the antidiabetic potential of AN, in vitro prebiotic activities of the extracts/sub-extracts on certain *Lactobacillus* strains were evaluated in the present study. Regarding GG and LP strains, ANM was the most active extract and generated the highest percentage of the increase in probiotic biomass. The activity of ANM was very similar to the results of the standards on the growth of GG. ANM showed significantly higher activity than the standards on the LP strain. The biomass increase in LR following extract treatment was not significant.

Recent in vivo studies showed that *Astragalus* polysaccharides had the potential to enhance insulin sensitivity by modifying gut microbiota in diabetic rats [50,51,52]. Besides the hypoglycemic effect of AST IV, it was further supported by modulation of intestinal flora and an elevation in butyric acid levels [26,53]. Consistent with the previously mentioned investigations, the methanol extract exhibited results comparable to the standards, which may be attributed to its relatively high saponin content and the possible synergistic effects of other secondary metabolites present in the total extract. These results provide initial insights into the prebiotic potential of AN extracts/sub-extracts; however, further studies are needed to explore their effects on intestinal microbiota more comprehensively and to determine their selectivity in promoting beneficial activity.

Antioxidant-rich plants are gaining increasing value in nutraceutical and pharmaceutical contexts associated with the management of degenerative and chronic health problems, including diabetes [54]. Hyperglycemic conditions, which induce oxidative stress through multiple pathways, play a significant role in the development of diabetes and the occurrence of complications. Managing oxidative stress in tissues may provide significant benefits in preventing diabetes and its complications [55]. Prior research indicated that polysaccharides, saponins, and flavonoids from the *Astragalus* acted as natural antioxidants. They can mitigate oxidative stress by directly eliminating free radicals, enhancing the efficacy of antioxidant enzymes, suppressing the activity of pro-oxidative enzymes, inhibiting lipid peroxidation, chelating metal ions, and modulating signaling pathways [56]. In the present study, assays for radical scavenging and reduction capacity were used to evaluate the antioxidant capabilities of the extracts/sub-extracts. ANC showed the highest antioxidant activity among all extracts/sub-extracts. These results were determined to align with the high flavonoid concentration in chloroform sub-extract (11.72 ± 0.01 mg QE). Additionally, ANH, which has a high flavonoid content compared to the other extracts/sub-extracts, exhibited the lowest activity in all experiments. Therefore, it may be suggested that the flavonoid content of the plant alone is not responsible for its antioxidant activity.

In the earlier research on *Astragalus* plants, which involved the antioxidant activity test of extracts/sub-extracts obtained from the roots and aerial organs utilizing solvents with varying polarity, a range of antioxidant activities was observed in these experiments. Notably, the highest antioxidant activity was observed in fractions, predominantly from non-polar extracts, which contained substantial phenolic content [57,58]. For instance, in the study of Kurt-Celep et al. (2021), methanol extracts *of A. campylosema* and *A. hirsutus* were evaluated using six different in vitro antioxidant activity methods. The test results indicated that leaf and flower extracts of *A. campylosema* and *A. hirsutus* exhibited more antioxidant activity than the roots, and the total flavonoid concentrations in the leaves and flowers were also higher [40]. In conclusion, the antioxidant activities observed in this study were largely consistent with previous findings regarding the relationship between flavonoids and antioxidant capacity.

In line with the global trend towards natural product-based drug discovery, this study combined in vitro and in silico techniques to examine the DPP IV, PTP1B, α-amylase, and AGEs-mediated mechanisms underpinning the antidiabetic effects of *Astragalus*, with a particular focus on five cycloartane saponins. A previous study demonstrated through pharmacophore modeling and molecular docking analyses that AST IV exhibited strong interactions with PTP1B, primarily via hydrogen bonding. The findings of the in vitro experiment confirmed that AST IV was a potent and selective inhibitor of PTP1B [43]. Although previous in silico studies examined that metabolic conditions, diabetes, and diabetes-related complications were linked to some *Astragalus* saponins, a comprehensive evaluation of diabetes-related pathways on astragalosides is lacking [59,60,61].

Both AutoDock and AutoDock Vina produced consistent results in the docking studies of the protein-ligand complexes in this study. The target proteins used in the in silico docking studies were chosen to maintain coherence with the in vitro assays and are frequently associated with diabetes-related mechanisms. ASTs II and III demonstrated the highest and most comparable docking scores among the tested ligands, indicating strong binding affinities for the target proteins and highlighting their potential as effective candidates for therapeutic development.

Ligand efficiency (LE), defined as the ratio of the binding energy to the number of heavy atoms in the ligand, provides valuable insights into the quality of ligand binding relative to its size. In this study, both ASTs II and III displayed superior ligand efficiencies compared to the other compounds. These ligands demonstrated a high degree of binding affinity per atom, indicating that they efficiently bound to their respective protein targets with minimal molecular complexity. This high ligand efficiency is particularly relevant for drug development, where smaller and more efficient molecules are often preferred for their ease of synthesis, fewer side effects, and better pharmacokinetics.

Additionally, the selectivity of ligands for their target proteins is crucial to their therapeutic potential. Docking studies revealed that AST II and AST III exhibited selective binding to the active sites of their respective targets, with strong interactions involving both hydrogen bonds and hydrophobic contacts. Their binding profiles suggest that these ligands can effectively discriminate between similar target sites, thus enhancing their potential as selective modulators for therapeutic applications.

The observed binding affinity trends for the protein-ligand pairs aligned with the established biological functions of the target proteins. ASTs II and III consistently showed the best docking scores and inhibition constants across all proteins, indicating their strong potential to inhibit these targets. For instance, in the case of 1J2E (DPP IV), AST II exhibited a docking score of −9.3 (AutoDock Vina) with an inhibition constant of 8.57 mM, suggesting moderate binding affinity. In contrast, AST III showed a comparable score and inhibition constant, highlighting the similarity between the binding profiles of these two ligands. Furthermore, ASTs I and IV had significantly lower docking scores and higher inhibition constants, further confirming that ASTs II and III were the most promising ligands among the groups. These trends emphasize that the chemical structure and size of ligands play a significant role in determining their binding affinities and effectiveness in inhibiting their target proteins. ASTs II and III appeared to have an optimal balance of size, flexibility, and binding efficiency, making them attractive candidates for further development.

Hydrogen bonds are vital in stabilizing ligand–protein binding by creating specific interactions between the donor and acceptor groups. For instance, in the case of AST I binding to 1J2E, hydrogen bonds were observed between the ligand and residues, such as LEU410, SER212, and VAL303, helping to anchor the ligand within the active site. Similarly, AST II and CEE formed hydrogen bonds with residues such as ARG358, GLU206, and GLU153, strengthening ligand-protein interactions.

In addition to hydrogen bonds, hydrophobic interactions are critical contributors to overall binding affinity. Hydrophobic residues, including TRP305, PHE364, and PRO159, frequently participated in interactions with ligands across the protein targets. These interactions help stabilize the hydrophobic core of the protein-ligand complex, enhancing binding stability and specificity. For example, ASTs II and III showed strong hydrophobic interactions with residues, such as VAL303, PHE364, and PHE280, further contributing to their superior binding profiles.

Notably, the combination of hydrogen bonding and hydrophobic interactions often results in a synergistic effect, where the ligands form multiple points of contact with the target proteins, leading to higher docking scores. This combination of interactions suggests that ASTs II and III may have more favorable and stable binding modes than the other ligands tested.

The docking studies presented herein highlighted the importance of both ligand efficiency and selectivity in determining the binding affinity of ligands to their target proteins. ASTs II and III emerged as the most promising ligands, exhibiting superior docking scores, strong hydrogen bonding, and hydrophobic interactions with the target proteins, whereas CCE demonstrated the lowest potential. The results were partly consistent with in vitro results. These results suggested that these ligands may serve as valuable lead compounds for future drug development targeting PTP-1B, α-amylase, DPP IV, and RAGE.

## 4. Materials and Methods

### 4.1. Chemicals

All enzymes, reagents, chemicals, and reference standards were of analytical grade or higher and were obtained from Sigma-Aldrich (St. Louis, MO, USA), unless otherwise stated. Protein tyrosine phosphatase 1B was supplied by Cayman Chemical Company (Ann Arbor, MI, USA). The standard compounds used included vildagliptin (SML2302), ursolic acid (U6753), quercetin (Q4951), acarbose (Y0000500), escin (E1378), gallic acid (G7384), caffeic acid (C0625), butylated hydroxytoluene (W218405), and astragalosides I–IV (SMB00318, SMB00316, SMB00317, and 74777, respectively). Kieselgel 60 (0.063–0.200 mm, Merck (Rahway, NJ, USA), 7734), Sephadex^®^ LH-20 (25–100 µm, Sigma-Aldrich), and 1 mm preparative TLC glass plates (Supelco, (Bellefonte, PA, USA), Z740216) were used for chromatographic analyses. Enzyme inhibition assays were carried out with protein tyrosine phosphatase 1B (Cayman Chemical, 10010896), dipeptidyl peptidase 4 (Sigma-Aldrich, D4943), and α-amylase (Sigma-Aldrich, A3176).

### 4.2. Plant Material

Roots of *A. noeanus* were collected from Mount Hazarbaba, Sivrice, Elazığ, Türkiye (38.418144, 39.304322), in July 2021. The botanical identification of the plant samples was conducted by the Assoc. Prof. Serpil Demirci Kayıran. A specimen voucher of the plant was preserved in the Herbarium of the Faculty of Pharmacy at Çukurova University (No: 1776).

### 4.3. Extraction and Isolation

The air-dried and powdered roots of *A. noeanus* (1 kg) were macerated overnight with hexane (4 L) under ambient conditions (ANH yield: 0.21%). The remaining plant material was macerated with methanol (4 L) for 3 days (9 h in an orbital shaker). This process was repeated three times. The combined methanol extracts were concentrated under reduced pressure to yield a crude extract (ANM yield: 11.3%). The total MeOH extract was dissolved in 500 mL of water and subsequently extracted with CHCl_3_ (4 × 200 mL) and *n*-BuOH (saturated with H_2_O) (4 × 200 mL) in a separatory funnel, respectively, to obtain CHCl_3_ (yield 0.94%), *n*-BuOH (yield 6.72%), and remaining water sub-extracts. An aliquot of the remaining water and all fractions were entirely evaporated using a rotary evaporator and freeze dryer (−80 °C, 48 h). All extracts were stored at +4 °C for subsequent analysis.

The *n*-BUOH sub-extract of *A. noeanus* was fractionated by a Sephadex LH-20 column (100 g) eluting with MeOH to yield six main fractions, namely A–F. A was subjected to normal-phase silica gel column chromatography (500 g) and eluted with a stepwise CHCl_3_/MeOH/H_2_O gradient (100:0:0−50:50:10, *v*/*v*/*v*), yielding 95 fractions. These fractions were then combined into 16 pooled fractions (A_1_–A_16_) based on their TLC profile similarities. The A_4–5_ fractions were subjected to PTLC and separated using a mobile phase consisting of CHCl_3_/MeOH/H_2_O (11:5:1, *v*/*v*/*v*). The bands were scraped off the plate based on their coloration after visualization with vanillin–sulfuric acid reagent. This procedure was repeated twice. Subsequently, semi-preparative HPLC (Agilent (Santa Clara, CA, USA) 1260 Infinity II LC System) eluting with a gradient mobile phase system (flow rate: 2.8 mL/min, 0–50 min: 10–40% MeCN/H_2_O, ACE 5 C_18_ column) led to the purification of CCE (3 mg), whose structure was confirmed by comparison of its NMR spectroscopic (Bruker (Billerica, MA, USA) Bruker Avance III 500 MHz) and LC-HRMS spectrometric (Thermo Scientific-Q Exactive (Waltham, MA, USA)) data with literature values. For activity tests, *A. noeanus* extracts/sub-extracts were prepared in methanol at a concentration of 1 mg/mL, and five saponins (ASTs I–IV and CCE) were prepared in methanol at 500 µg/mL.

### 4.4. In Vitro Diabetes-Related Bioactivity Methods

All biological activity assays were performed as described below. In this study, α-amylase, DPP-4, PTP1B, and AGEs inhibition assays were performed for both the extracts/sub-extracts and the saponins. In contrast, other biological activity assays, including antioxidant and prebiotic activity tests, were performed only for the extracts/sub-extracts, as these assays are indirectly related to antidiabetic mechanisms and were intended to assess the overall biological potential of the extracts/sub-extracts rather than the isolated saponins, for which only limited quantities were available. Other biological activity assays, including antioxidant and prebiotic activity tests, were carried out only for the extracts/sub-extracts. A two-stage screening strategy was adopted, consisting of an initial single-concentration primary screening (1 mg/mL for extracts; 500 µg/mL for saponins) followed by comparative evaluation using calibration curves of standard inhibitors to express activities as standard-equivalent inhibitory capacities. Some of the reference data used in the activity evaluation were shared with our previous publication [46], as both studies were derived from the same thesis project. However, the current study focuses on different plant materials and different sets of samples, and presents new datasets and extended analyses.

#### 4.4.1. α-Amylase Inhibition Activity

The α-amylase inhibition experiment was conducted utilizing 3,5-dinitrosalicylic acid (DNSA). 20 µL of α-amylase enzyme [10 units/mL in potassium phosphate monobasic (pH: 7) buffer] was combined with 20 µL of the extract and incubated for 10 min at 30 °C. Subsequent to the incubation period, 50 µL of the 10 mg/mL starch solution was introduced to each well and incubated for 15 min. The reaction was ended by the addition of 270 µL DNSA reagent and thereafter heated for 10 min in a water bath at 90 °C. The solution was cooled to room temperature and diluted with 1 mL of distilled water. The absorbance was measured at 540 nm. The blank and background solutions were prepared by replacing the sample and enzyme solutions, respectively, with an equivalent volume of buffer solution. Acarbose (31.25–1000 μg/mL) was utilized as the reference material, and the reaction was conducted in the same manner as with the plant extract described previously. The α-amylase inhibitory activity was quantified as a percentage of inhibition [62].

#### 4.4.2. Protein Tyrosine Phosphatase 1B (PTP1B) Inhibitory Assay

The PTP1B inhibitory activities of the plant extracts and saponins were assessed colorimetrically using p-nitrophenyl phosphate (*p*NPP) as the substrate. PTP1B reaction buffer was prepared with 50 mM citrate (pH 6.0), 0.1 M NaCl, 1 mM EDTA, and 1 mM DTT. In a well plate (final volume 100 µL), 40 µL of buffer-diluted PTP1B enzyme was introduced, either with or without a test sample. The plate was pre-incubated at 37 °C for 10 min, and subsequently, 50 µL of 2 mM *p*-NPP in PTP1B reaction buffer was introduced. After incubating at 37 °C for 15 min in darkness, the reaction was stopped by the addition of 10 M NaOH. The absorption value of the final solution was quantified at 405 nm using a spectrophotometer (Thermo Scientific™ Varioskan Sky). The nonenzymatic hydrolysis of 2 mM *p*-NPP was corrected by measuring the blank absorbance obtained in the absence of the enzyme. Tests were replicated 3 times. Ursolic acid (31.25–1000 μg/mL) was used as a positive control, and the results were presented as percent inhibition [46].

#### 4.4.3. Dipeptidyl Peptidase IV (DPP IV) Inhibitory Activity

The in vitro DPP IV inhibition assay was conducted as previously indicated by Chakrabarti et al. (2011), with minor modifications [3]. 35 µL of the extracts in 1 mg/mL, saponins in 500 μg/mL, and vildagliptin, as a positive control, in 1000 to 31.25 μg/mL concentrations were applied in 96-well microplates. 15 µL of a DPP IV enzyme solution (0.05 units/mL in Tris HCl buffer, pH 7.5) was put into micro well plates and incubated for 15 min at 37 °C. 50 µL of substrate GPPN (0.2 mM) was added to the resulting mixture and thereafter incubated for 30 min at 37 °C. 25 µL of 25% glacial acetic acid was introduced to the mixture to terminate the process. The final volume in each well of the plate is 125 µL. The amount of released *p*-nitroanilide was measured by reading the absorbance at 405 nm using a microplate reader. The tests were conducted three times. The results were expressed as a percentage of the inhibitory activity of extracts, saponins, and the standard.

#### 4.4.4. Advanced Glycation End Products (AGEs) Assay

The BSA-glucose reaction model system was utilized in order to test the inhibitory activity on glycation of standard and samples. The experiment was conducted using the method that was utilized before by Barak et al. (2022). 1 mL of bovine serum albumin (BSA, 10 mg/mL) in phosphate buffer (0.1 M, pH 7.4) and 1 mL of 0.5 M glucose were combined with 1 mL of samples or standard (1 mg/mL). 1 mL of phosphate buffer, 1 mL of BSA solution, and 1 mL of glucose solution were used to prepare the blank solution. An equivalent volume of buffer was added instead of the sample solutions to prepare the control solution. Quercetin was utilized as reference material. After the reaction mixtures were prepared and enclosed with a tight cap, they were placed in a water bath at a temperature of 55 °C and shaken at a rate of 100 rpm. After that, the mixtures were allowed to cool down to room temperature. The fluorescence intensity was measured using the Varioskan^TM^ LUX multimode microplate reader (Thermo Scientific ^TM^) in the 370 nm excitation/440 nm emission band after the 40 h incubation period [63].

#### 4.4.5. Prebiotic Activity

Three distinct *Lactobacillus* species were employed to assess the biomass augmentation influenced by plant extracts. MRS broth is used as a pure medium to assess the growth rates of microorganisms. PreBIULIN FOS (Gobiotics, Hoogerheide, The Netherlands), Biolin-P (Gobiotics, The Netherlands), and glucose were used as the positive control for the study. The extracts/sub-extracts selected for the study were ANM, ANB, and ANW, all of which are water-miscible. Each active substance examined was inserted into 10 mL of MRS broth at a concentration of 1% (*w*/*v*). *Lactobacillus rhamnosus* GG, *Lactobacillus reuteri*, and *Lactobacillus paracasei* were introduced into MRS broth for a 24 h incubation. Following this period, 1% of these cultures were separated and inoculated into MRS broth media supplemented with 1% of either prebiotic samples, plant extracts/sub-extracts, or glucose. The test tubes were subsequently put in incubators with 5% CO_2_ and incubated for 24 h. Upon completion of incubation, samples were taken from the tubes, and cell biomass was quantified using a U*V*/*V*IS spectrophotometer (Thermo Scientific Evolution 201/220) at OD_600_ [64].

### 4.5. In Vitro Investigation of Antioxidant Potential

#### 4.5.1. DPPH Radical Scavenging Activity

The DPPH radical-scavenging assay was performed following the previously established protocol. 200 μL of freshly made DPPH solution was mixed with 25 μL of the samples and butylated hydroxytoluene (31.25–1000 μg/mL). The mixture was incubated in the absence of light at room temperature for 30 min. The absorbance was measured at 517 nm, utilizing methanol as a blank reference. All measurements were conducted in triplicate. The percent of antioxidant activity for DPPH radical scavenging was determined by comparing the absorbance of the samples to that of the control [65].

#### 4.5.2. Ferric Reducing Antioxidant Power (FRAP)

The capacity of the AN extracts/sub-extracts to reduce ferric ions was determined by applying the method used previously [66]. The FRAP reagent was prepared just before use by combining TPTZ [2,4,6-tris(2-pyridyl)-s-triazine] with FeCl_3_ in an acetate buffer. Following a 30-min incubation at 37 °C in the dark, 10 μL of the sample or reference was combined with 260 μL of FRAP reagent and incubated under the same conditions. The absorbance was measured at 593 nm using the plate reader. The results were given as mM FeSO_4_ per g of extract. All determinations were conducted in triplicate.

#### 4.5.3. Cupric Reducing Antioxidant Capacity (CUPRAC)

The cupric ion-reducing ability of the extracts was assessed using a previously applied method by Barak et al. (2023). Equal volumes (85 μL) of freshly prepared CuSO_4_, ethanolic neocuproine, and ammonium acetate buffer were combined. Extracts and standard dilutions were mixed with the reaction mixture. After a 1 h incubation period at room temperature, the absorbance was measured at 450 nm using the microplate reader. The results were presented as mg of ascorbic acid equivalent per gram of extract [67].

#### 4.5.4. Total Antioxidant Capacity

In order to determine the total antioxidant capacity of the extracts/sub-extracts, the spectrophotometric method used by Barak et al. (2024) was employed. A reaction mixture including sodium phosphate monobasic (28 mM), sulfuric acid (0.6 M), and ammonium molybdate (4 mM) was introduced into vials with the plant extracts. The vials were securely covered and put in a 95 °C water bath for a 90-min incubation procedure. The mixtures were transferred to a 96-well plate when reaching ambient temperature. The absorbance of the samples was quantified at 695 nm with a plate reader. The total antioxidant capacity of the samples was expressed as milligrams of ascorbic acid equivalent per gram of dry extract [68].

### 4.6. Chemical Profile Assessment

#### 4.6.1. Total Flavonoid Content

Total flavonoid content (TFC) was measured by a colorimetric assay. 50 μL of each sample was inserted into a solution of 10% aluminum chloride and 1 M sodium acetate in equal amounts. The mixture was incubated for 30 min at ambient temperature in the dark. The absorbance was measured at 415 nm using a plate reader. The calibration curves were generated using a quercetin solution. The results were presented as mg of quercetin equivalents (QE) per g of dry extract. Measurements were conducted in triplicate [69].

#### 4.6.2. Total Saponin Content

The concentration of total saponins (TPS) was measured by the method described by Lim et al. (2020). Simply, 0.25 mL of the sample was added to 1 mL of the reagent mixture containing glacial acetic acid/sulfuric acid 1:1 *v*/*v*. Each tube was vortexed and allowed to react for 30 min before the absorbance reading at 527 nm in a plate reader (Multiskan Sky, Thermo Scientific). Escin was utilized as a reference substance with a concentration range of 0–1000 μg/mL. Saponin content was quantified as g of escin equivalents. Each estimation was performed in triplicate [70].

#### 4.6.3. HPTLC Quantification of Saponins

The concentrations of ASTs I-IV in the extracts/sub-extracts were determined with the method conducted previously by Li et al. (2020). The concentration of the standard solutions (100 µg/mL) was prepared in methanol. Similarly, 1 mg and 10 mg of AN extracts/sub-extracts were dissolved in 1 mL of methanol. To filter the extracts/sub-extracts, a syringe filter with a pore size of 0.45 µm was utilized. Samples in varying quantities, ranging from 15 to 40 µL, were administered in triplicate. Each application of the standard mixture ranged from 2 µL to 12 µL. For astragalosides, the mobile phase had a volume ratio of CHCl_3_/MeOH/EtOAc/H_2_O (13:7:2:2). The mobile phase of the cyclocanthoside E was determined as CHCl_3_/MeOH/H_2_O (11:5:1). The band length of the applications was 8 mm on silica gel aluminum HPTLC plates 60 F254, utilizing the Camag Automatic TLC Sampler IV (Camag, Muttenz, Switzerland). Developments were conducted in the Camag Automatic Developing Chamber (ADC-2). The saturation procedure endured for 20 min prior to development. The humidity control is administered by ADC-2 utilizing MgCl_2_ (33% RH) for 15 min. The development level of the plates was set at 80 mm. Following derivatization with a 10% sulfuric acid in ethanol solution, densitometric analysis was conducted with the Camag TLC Scanner IV at 366 nm and Vision CATS software in absorption mode. The slit dimensions were defined as 5 × 0.2 mm, and the scanning speed was 20 mm/s. The quantities of standards in the extracts/sub-extracts were determined by comparing the AUCs with the calibration curve of the standards. The correlation coefficient (R) of the calibration curves exceeded 0.998. The presence and amounts of standard chemicals in the extracts/sub-extracts were confirmed by comparing the retention factors (R*f*: AST I = 0.532, AST II = 0.417, AST III = 0.345, AST IV = 0.292, CCE = 0.326) and the overlapped UV spectra of each extract/sub-extract with the standards [71].

### 4.7. Molecular Docking Studies

Docking studies were conducted using AutoDock [72] and AutoDock Vina [73,74] software to analyze the binding interactions of the selected drug molecules (ASTs I-IV and CCE) with key diabetes-related protein targets. The crystallographic structures of the proteins were retrieved from the Protein Data Bank (PDB) database [75]: PTP-1B (PDB codes 1NNY and 1T49), porcine pancreatic α-amylase (PDB code 1OSE), DPP IV (PDB code 1J2E), and the receptor for advanced glycation end-products (RAGE, PDB code 3O3U).

Proteins were prepared using AutoDockTools, which involved removing water molecules, adding hydrogen atoms, assigning partial charges, and identifying the active binding sites. To ensure the reliability of the docking results, the protein structures were energy-minimized using NAMD software [76] in combination with the CHARMM force field [77] prior to docking. The presented figures were prepared using AutoDockTools for 3D and LigPlot [78] for 3D representations. For each protein-ligand complex, both blind docking and active site docking were performed to explore the possible binding modes. In the docking simulations, the ligands were treated as flexible to allow conformational adaptability during docking, whereas the protein structures were kept rigid. These methods were employed to predict the binding affinity and interactions between ligands and target proteins.

Although the present in vitro and in silico results suggest promising antidiabetic potential for *Astragalus noeanus*, these findings should be regarded as preliminary. Further in vivo and clinical studies are required to confirm the efficacy, safety, and pharmacokinetic behavior of the identified bioactive compounds and to validate their relevance in physiological glycemic control.

### 4.8. Statistics

Data were expressed as mean ± SD of triplicate independent experiments. One-way ANOVA was used to compare group means (α = 0.05). Normality and variance homogeneity were verified (Shapiro–Wilk and Brown–Forsythe tests). Post hoc comparisons were performed using Sidak’s multiple-comparison test for all pairwise analyses or Dunnett’s test for multiple-versus-control comparisons, as appropriate. All analyses were carried out using GraphPad Prism version 8.0.1 (GraphPad Software, San Diego, CA, USA).

## 5. Conclusions

This study provides novel insights into the antidiabetic potential of *Astragalus noeanus* extracts/sub-extracts, identifying them as promising sources of bioactive phytochemicals. Both the crude extracts and five cycloartane-type saponins exhibited significant activity against a range of diabetes-related targets in vitro and in silico. These findings expand the pharmacological profile of the *Astragalus* genus, with *A. noeanus* emerging as a noteworthy candidate. The results suggest a synergistic contribution of the plant’s primary and secondary metabolites to its antidiabetic effects through multiple mechanisms. Further research is needed to clarify the underlying pathways, particularly those involving astragalosides IV and III, in the context of their therapeutic potential for diabetes management.

In conclusion, this study provides the first mechanistic evidence supporting the potential antidiabetic properties of *A. noeanus* based on in vitro and in silico analyses. However, these results represent an initial step, and comprehensive in vivo investigations are essential to substantiate the observed effects and translate them into pharmacological relevance.

## Figures and Tables

**Table 1:** HPTLC results for astragalosides I, II, III, and IV and cyclochantoside E amounts of *Astragalus noeanus* root extracts/sub-extracts.

Extract/ Sub-Extract	(μg/mg)				
	AST I	AST II	AST III	AST IV	CCE
ANM	trace	trace	ND	14.28 ± 1.29	117.27 ± 6.71
ANC	ND	ND	ND	ND	trace
ANB	trace	3.5 ± 0.07	ND	31.25 ± 2.63	197.13 ± 12.49
ANW	trace	trace	ND	1.44 ± 0.09	trace
ANH	ND	ND	ND	ND	ND

ND: not detected. ANM: A. noeanus methanol extract, ANB: A. noeanus butanol sub-extract, ANW: A. noeanus water sub-extract.

**Table 2:** α-amylase, PTP1B, DPP IV, and AGEs inhibitory activities of *A. noeanus* extracts and standard molecules.

*A.* noeanus Extracts/ Sub-Extracts **	ANM *	ANB	ANC	ANW	ANH	Standard Inhibitors	
						1000 μg/mL	500 μg/mL
% α-amylase Inhibition	91.00 ± 0.18	82.84 ± 0.04	84.99 ± 0.92	93.49 ± 0.06	79.31 ± 0.05	92.40 ^#^ ± 0.07	88.89 ^#^ ± 0.01
% PTP1B Inhibition	84.86 ± 0.00	66.80 ± 0.04	77.00 ± 0.03	85.93 ± 0.04	64.92 ± 0.07	93.62 ^##^ ± 0.07	90.75 ^##^ ± 0.07
% DPP IV Inhibition	80.81 ± 0.00	76.23 ± 0.01	62.48 ± 0.00	87.26 ± 0.00	58.85 ± 0.01	98.46 ^§^ ± 0.07	93.21 ^§^ ± 0.01
% AGEs Inhibition	91.38 ± 0.15	76.95 ± 4.66	60.83 ± 13.61	83.70 ± 5.20	69.09 ± 12.25	93.82 ^§§^ ± 6.62	85.44 ^§§^ ± 6.88

* Results were expressed as the mean of triplicates and standard deviation; ** Extracts/sub-extracts were used in 1 mg/mL concentration. Each analysis included the standard reference inhibitor at different concentrations (31.25–1000 µg/mL); ANM: *A. noeanus* methanol extract. ANB: *A. noeanus* sub-butanol extract. ANC: *A. noeanus* sub-chloroform extract. ANW: *A. noeanus water* sub-extract. ANH: *A. noeanus* hexane extract; ^#^ Acarbose. ^##^ Ursolic Acid. ^§^ Vildagliptin. ^§§^ Quercetin.

**Table 3:** Results of *Astragalus* saponins and standard *α*-amylase, PTP1B, DPP IV, and AGEs inhibition activities *.

**	AST I	AST II	AST III	AST IV	CCE	Standard Inhibitors	
						1000 μg/mL	500 μg/mL
% *α*-amylase Inhibition	85.59 ± 1.15	86.87 ± 0.30	89.26 ± 0.30	99.51 ± 0.59	82.71 ± 0.04	92.40 ^#^ ± 0.07	88.89 ^#^ ± 0.01
% PTP1B Inhibition	70.24 ± 0.02	68.93 ± 0.06	77.58 ± 0.04	88.37 ± 0.01	66.60 ± 0.06	93.62 ^##^ ± 0.07	90.75 ^##^ ± 0.07
% DPP IV Inhibition	92.32 ± 0.01	80.09 ± 0.5	95.21 ± 0.00	96.77 ± 0.00	90.92 ± 0.00	98.46 ^§^ ± 0.07	93.21 ^§^ ± 0.01
% AGEs Inhibition	74.68 ± 1.00	72.78 ± 1.35	77.21 ± 2.05	82.14 ± 6.05	63,35 ± 0.90	93.82 ^§§^ ± 6.62	85.44 ^§§^ ± 6.88

* Results were expressed as the mean of triplicates and standard deviation; ** Saponins were used in a 500 µg/mL concentration. Each analysis included the standard reference inhibitor at different concentrations (31.25–1000 µg/mL); ASTI: astragaloside I, ASTII: astragaloside II, ASTIII: astragaloside III, ASTIV: astragaloside IV, CCE: cyclocanthoside E; ^#^ Acarbose. ^##^ Ursolic Acid. ^§^ Vildagliptin. ^§§^ Quercetin.

**Table 4:** In vitro prebiotic activities of *A. noeanus* extracts.

*	MRS	GLU	BIO	PRE	ANM	ANB	ANW
**GG**	1.772 ^a^ ± 0.051	2.071 ^b^ ± 0.022	2.016 ^b^ ± 0.036	2.028 ^b^ ± 0.109	2.145 ^b^ ± 0.022	2.035 ^b^ ± 0.017	2.103 ^b^ ± 0.011
**LP**	1.587 ^b^ ± 0.038	1.535 ^bc^ ± 0.026	1.433 ^c^ ± 0.099	1.304 ^d^ ± 0.015	1.714 ^a^ ± 0.033	1.640 ^ab^ ± 0.014	1.625 ^ab^ ± 0.004
**LR**	1.608 ^ab^ ± 0.033	1.538 ^a^ ± 0.078	1.63 ^ab^ ± 0.061	1.638 ^ab^ ± 0.047	1.688 ^ab^ ± 0.057	1.567 ^ab^ ± 0.023	1.716 ^b^ ± 0.071

* Results were stated as the mean of triplicates ± standard deviation (S.D.) and absorbance values for cell density after 24 h; Microorganisms: GG: *Lactobacillus rhamnosus* GG. LR: *Lactobacillus reuteri*. LP: *Lactobacillus paracasei*; Standard commercial prebiotics: BIO (inulin. alpha-glucan oligosaccharide) and PRE (inulin. fructose); Abbreviations: GLU: glucose, MRS: medium, ANM: *A. noeanus* methanol extract, ANB: *A. noeanus* butanol sub-extract, ANW: *A. noeanus* water sub-extract. Superscript letters (a–d) indicate statistically significant differences among samples in the same row according to one-way ANOVA (*p* < 0.05).

**Table 5:** In vitro antioxidant activities of *A. noeanus* extracts.

Analysis	ANM	ANB	ANC	ANW	ANH
TOAC ^A^	105.06 ^a^ ± 10.23	100.32 ^a^ ± 1.52	107.71 ^a^ ± 7.32	18.58 ^b^ ± 0.21	ND.
FRAP ^B^	0.18 ^a^ ± 0.00	0.14 ^b^ ± 0.00	0.35 ^c^ ± 0.03	0.05 ^d^ ± 0.00	ND.
CUPRAC ^C^	39.01^a^ ± 1.31	34.68 ^b^ ± 1.08	65.61 ^c^ ± 5.69	53.58 ^d ^ ± 7.46	18.89 ^e^ ± 1.7
DPPH ^D^	17.85 ^a^ ± 0.21	12.00 ^b^ ± 0.19	44.34 ^c^ ± 0.15	39.02 ^d^ ± 0.32	ND.

Superscript letters (a–e) indicate statistically significant differences among samples in the same row according to one-way ANOVA (*p* < 0.05), ND = non-detected, TOAC: Total Antioxidant Capacity; FRAP: Ferric Reducing Antioxidant Power; CUPRAC: Cupric Reducing Antioxidant Capacity; DPPH: 2,2-diphenyl-1-picrylhydrazyl Radical Scavenging Assay; ^A^ The results were stated as the mean of triplicates ± standard deviation (S.D.) and as mg ascorbic acid equivalents (AAE) in a 1 g sample; ^B^ The results were stated as the mean of triplicates ± standard deviation (S.D.) and as Mm FeSO_4_ equivalents (FeE) in a 1 g sample; ^C^ The results were stated as the mean of triplicates ± standard deviation (S.D.) and as mg ascorbic acid equivalents in a 1 g sample; ^D^ The results were stated as the mean of triplicates ± standard deviation (S.D.) and as mg BHT equivalents in 1 a g sample; ANM: *A. noeanus* methanol extract, ANB: *A. noeanus* butanol sub-extract, ANC: *A. noeanus* chloroform sub-extract, ANW: *A. noeanus* water sub-extract, ANH: *A. noeanus* hexane extract.

**Table 6:** Docking scores of the ligands on the given proteins as computed by Autodock and Autodock-Vina. The inhibition constants (Ki) are given in micromolar (μM) or millimolar (mM).

		1J2E		1NNY		1OSE		1T49		3O3U		TOTAL
		Autodock	Vina	Autodock	Vina	Autodock	Vina	Autodock	Vina	Autodock	Vina	
AST I	DS	−4.34	−8.9	−2.41	−7.7	−3.32	−8.4	−1.92	−6.1	−6.02	−10.5	−56.29
	Ki	656.63 μM		17.13 mM		3.71 mM		39.33 mM		38.51 μM		
AST II	DS	−4.83	−9.3	−2.82	−7.4	−2.77	−8.1	−1.65	−6.3	−5.77	−10.1	−59.04
	Ki	287.41 μM		8.57 mM		9.38 mM		61.71 mM		59.07 μM		
AST III	DS	−4.10	−10.0	−3.14	−7.7	−3.16	−9.0	−1.68	−6.1	−5.22	−10.6	−60.70
	Ki	989.76 μM		5.01 mM		4.81 mM		59.08 mM		148.43 μM		
AST IV	DS	−3.75	−8.7	−2.93	−7.6	−2.50	−8.4	−1.09	−5.4	−5.88	−10.5	−56.75
	Ki	1.78 mM		7.10 mM		14.76 mM		158.03 mM		48.83 μM		
CCE	DS	−2.24	−9.2	−2.17	−7.5	−4.76	−8.5	+0.22	−5.7	−5.16	−10.6	−55.61
	Ki	22.63 mM		25.69 mM		323.82 μM		-		165.94 μM		

**Table 7:** Protein-ligand interactions for the given protein-ligand pairs. See Figure 5, Figure 6, Figure 7, Figure 8 and Figure 9 and Figures S6–S12 for graphical representations.

Protein-Ligand Pair	H-Bonding Interactions	Hydrophobic Interactions
1J2E-AST I	LEU410, LEU214, SER212, VAL303, THR304	ALA409, GLU408, ALA306, PRO218, TRP305, PRO159, TRP216, THR156, HSD363, GLU361, TRP215, PRO362, TRP305, TRP216
1J2E-AST II	ARG358, GLU206, GLU205, TYR547, ARG125	TYR666, VAL207, SER209, PHE357, PHE208, TYR585, SER552
1J2E-AST III	TRP305, VAL303, THR304, TRP216, ARG61, TRP157, SER106	PRO218, PRO159, LYS463, ILE63, TRP62, GLU361, SER217, TRP215, THR156, ILE107
1J2E-ASTIV	SER212, TRP305, PRO362, HSD363, PHE364	LEU477, LYS512, HSD533, LEU504, PRO475, ARG453, ASP501, PHE559, GLN505, GLY476, PRO510, MET509, SER511, PRO532
1J2E-CEE	LEU214, SER212, TRP215, VAL303, THR304, PRO362, ALA306, PHE364, TRP305	GLY183, GLY240, GLY183, HSD363
1NNY-AST I	LEU410, LEU214, SER212, VAL303, THR304	ALA409, GLU408, ALA306, PRO218, TRP305, PRO159, TRP216, THR156, HSD363, GLU361, TRP215, PRO362, TRP305, TRP216
1NNY-AST II	ARG358, GLU206, GLU205, TYR547, ARG125	TYR666, VAL207, SER209, PHE357, PHE208, TYR585, SER552
1NNY-AST III	TRP305, VAL303, THR304, TRP216, ARG61, TRP157, SER106	PRO218, PRO159, LYS463, ILE63, TRP62, GLU361, SER217, TRP215, THR156, ILE107
1NNY-AST IV	SER212, TRP305, PRO362, HSD363, PHE364	LEU477, LYS512, HSD533, LEU504, PRO475, ARG453, ASP501, PHE559, GLN505, GLY476, PRO510, MET509, SER511, PRO532
1NNY-CEE	LEU214, SER212, TRP215, VAL303, THR304, PRO362, ALA306, PHE364, TRP305	GLY183, GLY240, GLY183, HSD363
1OSE-AST I	LEU410, LEU214, SER212, VAL303, THR304	ALA409, GLU408, ALA306, PRO218, TRP305, PRO159, TRP216, THR156, HSD363, GLU361, TRP215, PRO362, TRP305, TRP216
1OSE-AST II	ARG358, GLU206, GLU205, TYR547, ARG125	TYR666, VAL207, SER209, PHE357, PHE208, TYR585, SER552
1OSE-AST III	TRP305, VAL303, THR304, TRP216, ARG61, TRP157, SER106	PRO218, PRO159, LYS463, ILE63, TRP62, GLU361, SER217, TRP215, THR156, ILE107
1OSE-AST IV	SER212, TRP305, PRO362, HSD363, PHE364	LEU477, LYS512, HSD533, LEU504, PRO475, ARG453, ASP501, PHE559, GLN505, GLY476, PRO510, MET509, SER511, PRO532
1OSE-CEE	LEU214, SER212, TRP215, VAL303, THR304, PRO362, ALA306, PHE364, TRP305	GLY183, GLY240, GLY183, HSD363

## Data Availability

The data is contained within the article and the Supplementary Materials.

## Supplementary Materials

The following supporting information can be downloaded at: https://www.mdpi.com/article/10.3390/ph19030352/s1, Figures S1–S5: NMR Spectra, Figures S6–S13: Docking Results, Table S1: Percent inhibition values of the controls.

## Abbreviations

The following abbreviations are used in this manuscript:

AGEs	Advanced Glycation End Products
AN	*Astragalus noeanus*
ANB	*Astragalus noeanus* Butanol Sub-Extract
ANC	*Astragalus noeanus* Chloroform Sub-Extract
ANH	*Astragalus noeanus* Hexane Extract
ANM	*Astragalus noeanus* Methanol Extract
ANW	*Astragalus noeanus* Water Sub-Extract
ASTI–IV	Astragaloside I–IV
BHT	Butylated Hydroxytoluene
BSA	Bovine Serum Albumin
CCE	Cyclocanthoside E
COSY	Correlation Spectroscopy
CUPRAC	Cupric Reducing Antioxidant Capacity
DM	Diabetes Mellitus
DNSA	3,5-Dinitrosalicylic Acid
DPP IV (DPP4)	Dipeptidyl Peptidase IV
EDTA	Ethylenediaminetetraacetic Acid
FRAP	Ferric Reducing Antioxidant Power
FeE	FeSO_4_ Equivalents
GIP	Glucose-Dependent Insulinotropic Polypeptide
GLP-1	Glucagon-Like Peptide 1
GG	*Lactobacillus rhamnosus* GG
HMBC	Heteronuclear Multiple Bond Correlation
HPTLC	High-Performance Thin-Layer Chromatography
HRMS	High-Resolution Mass Spectrometry
HSQC	Heteronuclear Single Quantum Coherence
IC_50_	Half-Maximal Inhibitory Concentration
LE	Ligand Efficiency
LP	*Lactobacillus paracasei*
LR	*Lactobacillus reuteri*
MRS	De Man, Rogosa, and Sharpe Medium
NMR	Nuclear Magnetic Resonance
OD_600_	Optical Density at 600 nm
PTP1B	Protein Tyrosine Phosphatase 1B
QE	Quercetin Equivalent
RAGE	Receptor for Advanced Glycation End Products
S.D.	Standard Deviation
TFC	Total Flavonoid Content
TOAC	Total Antioxidant Capacity
TPS	Total Saponin Content
TPTZ	2,4,6-Tris(2-pyridyl)-S-triazine
U*V*/*V*IS	Ultraviolet-Visible Spectrophotometry
pNPP	Para-Nitrophenyl Phosphate
