# Supplementary material for: Cycloartane-Type Saponins, Phytochemical-Rich Extracts, and Sub-Extracts from Astragalus noeanus Boiss. Exhibit In Vitro and In Silico Effects on Glucose Metabolism
Source: Pharmaceuticals (Basel). 2026 Feb 25;19(3):352. doi: 10.3390/ph19030352 (PMC13028877; doi:10.3390/ph19030352)
Supplement: Supplementary file 1 [file pharmaceuticals-19-00352-s001.zip › pharmaceuticals-3932902-supplementary.pdf]

Supporting Information

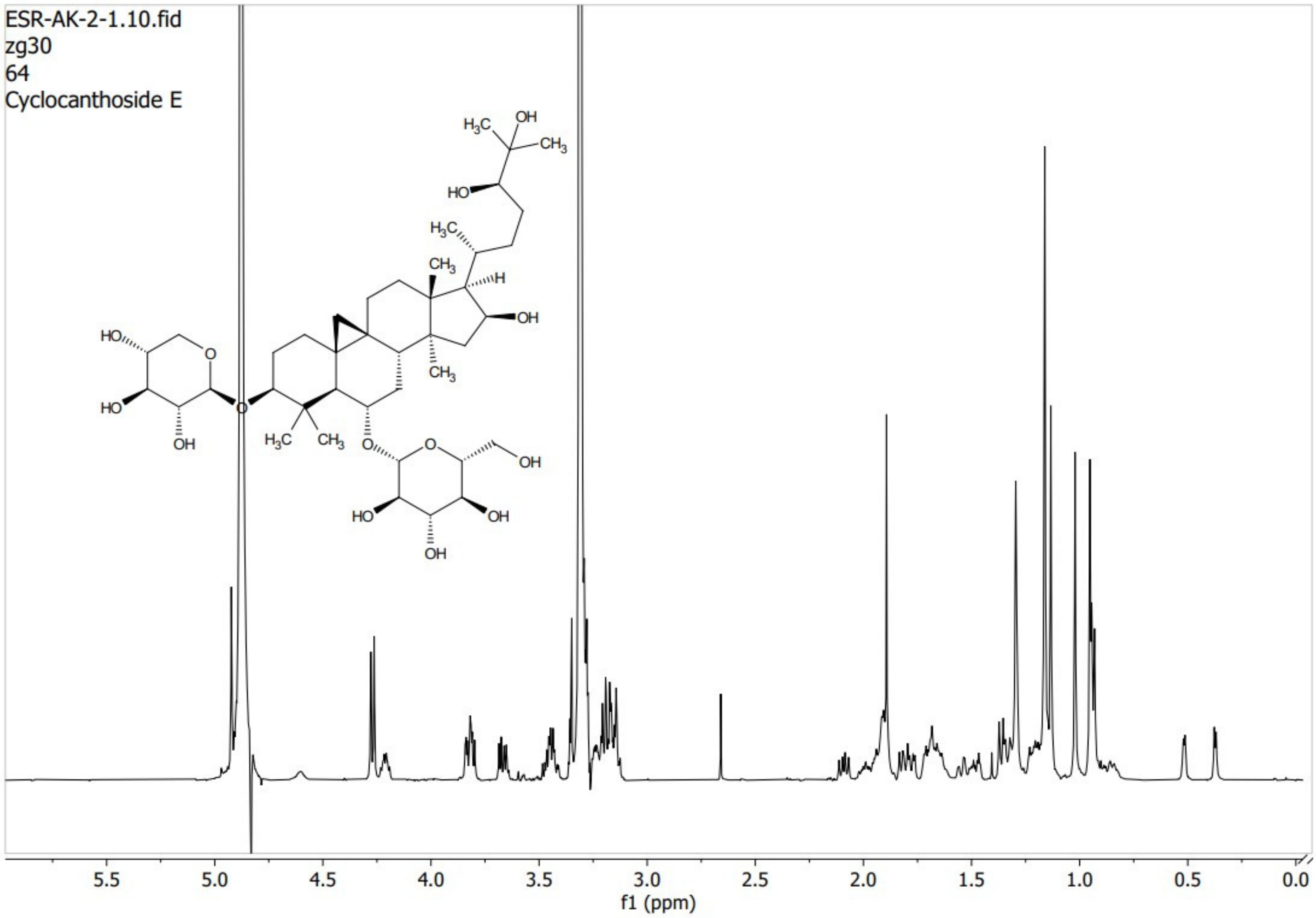

Figure S1: AN1 ^{1}H NMR spectra

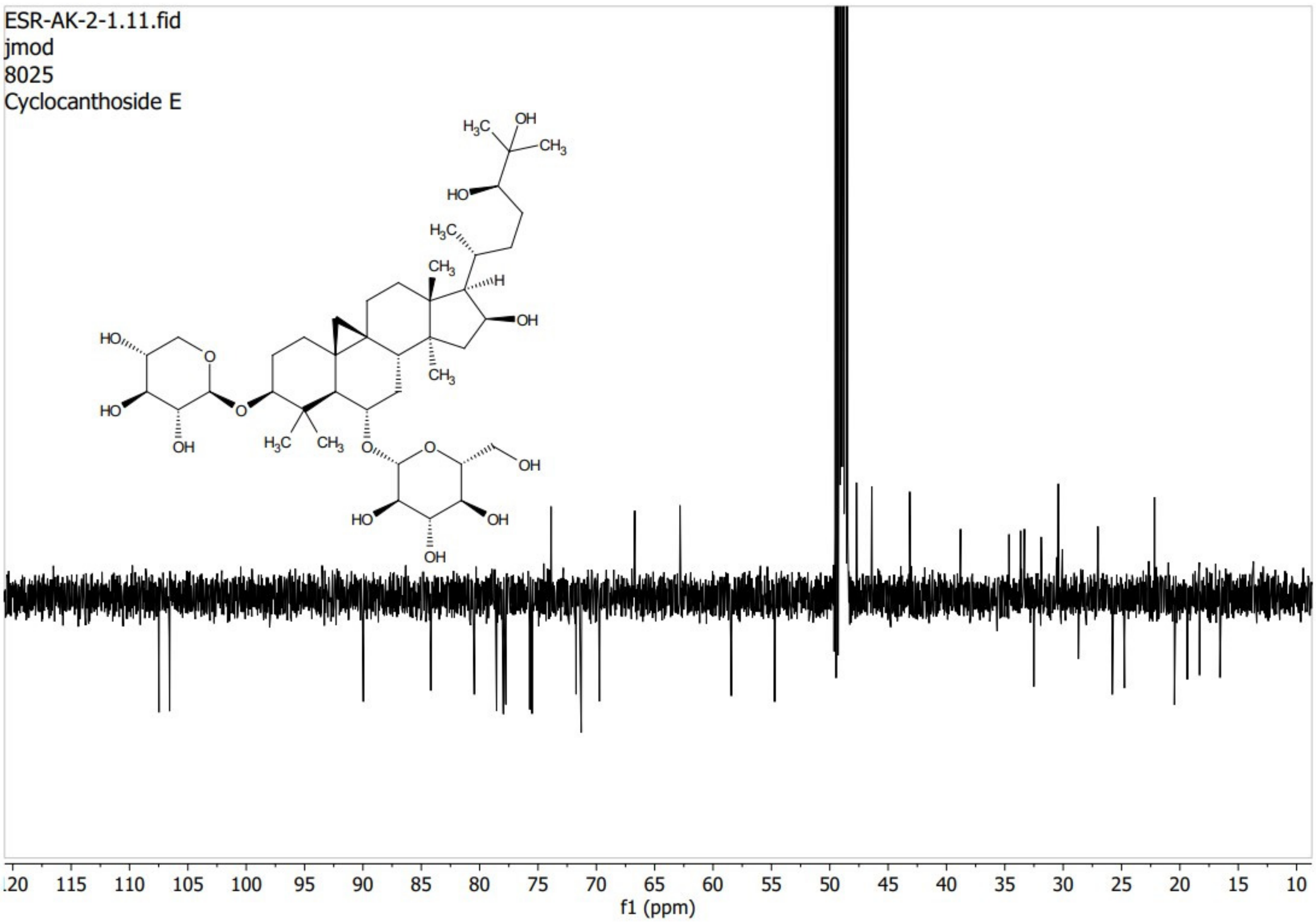

Figure S2: AN1 ^{13}C NMR spectra

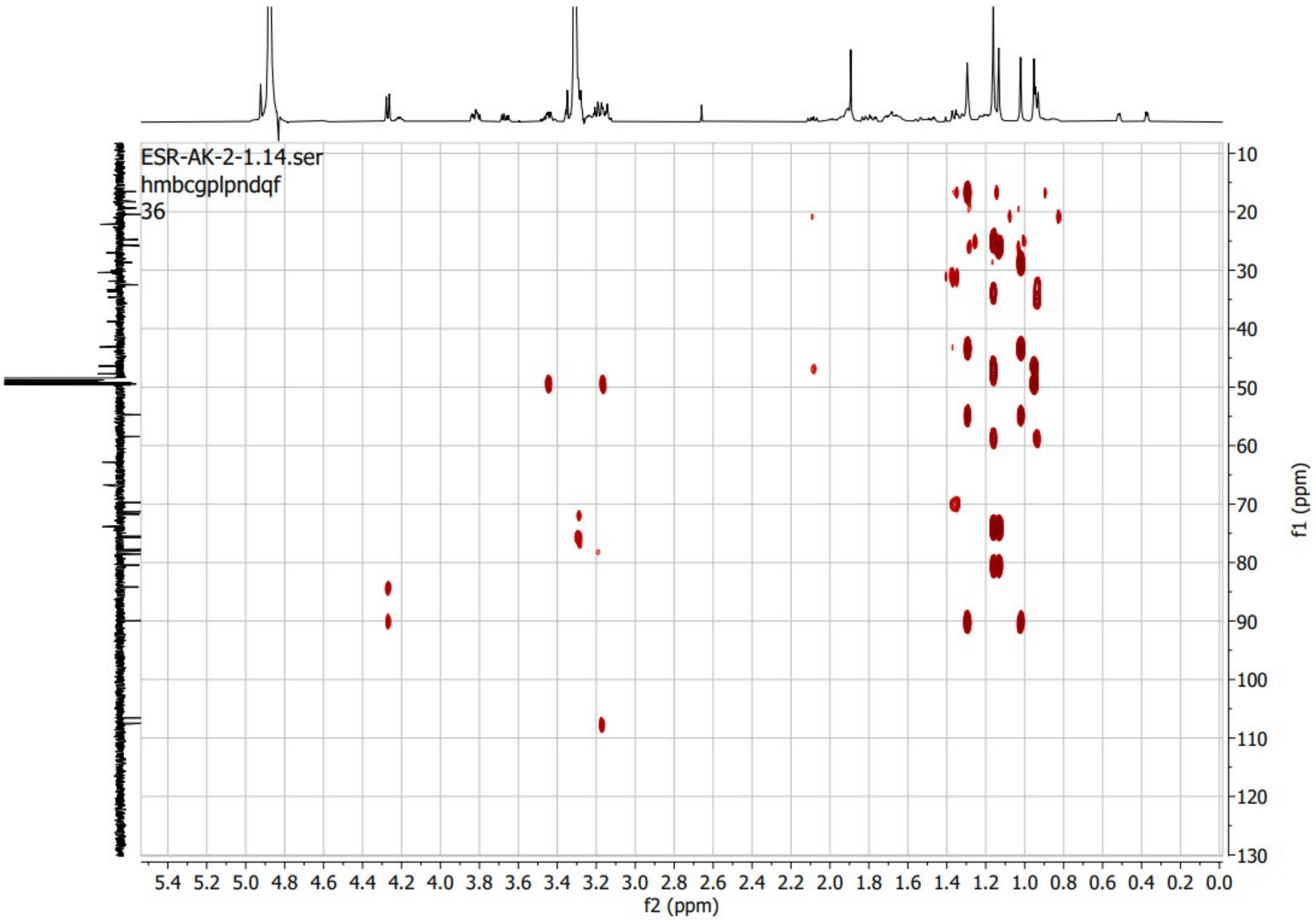

Figure S3: AN1 HMBC spectra

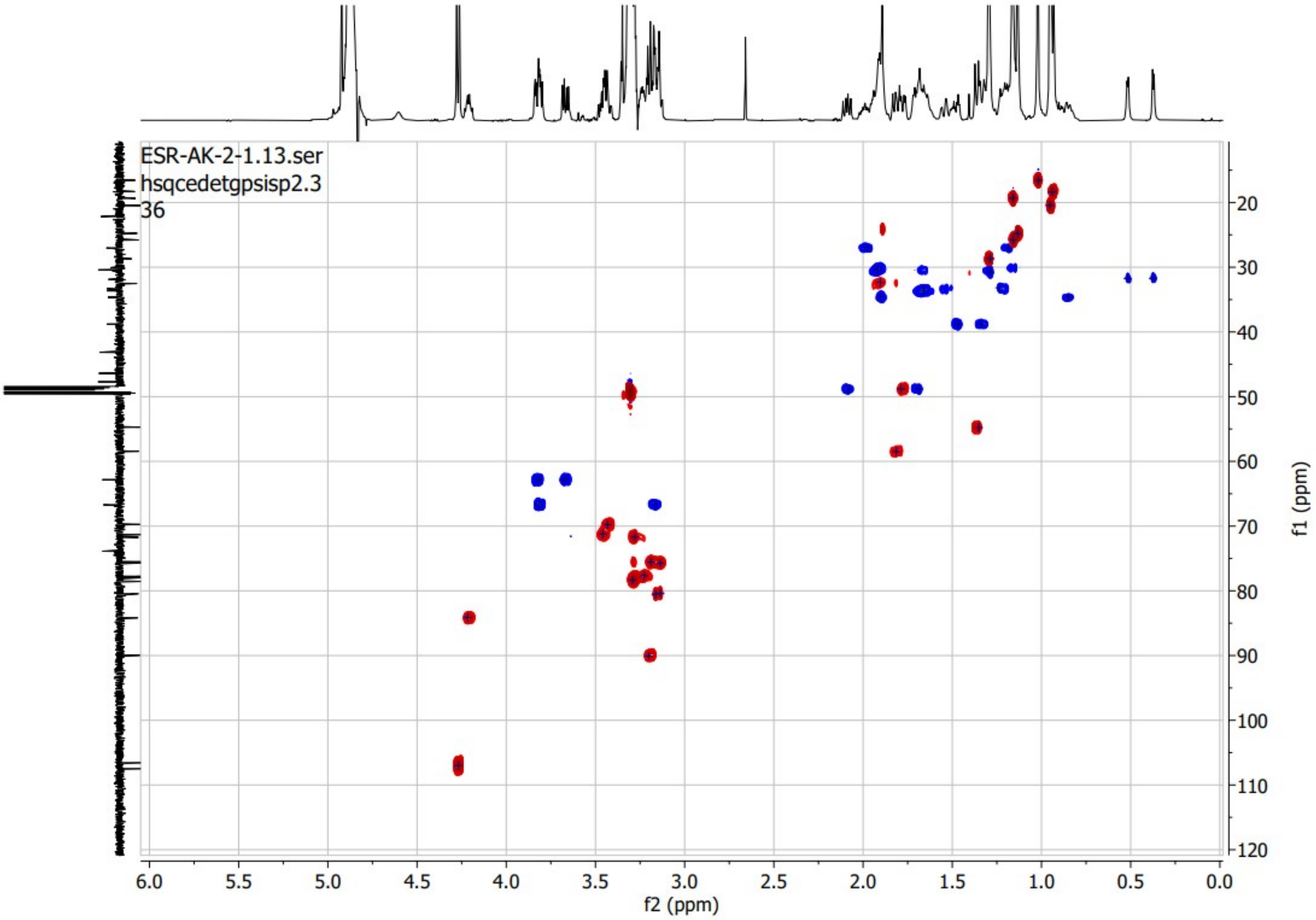

Figure S4: AN1 HSQC spectra

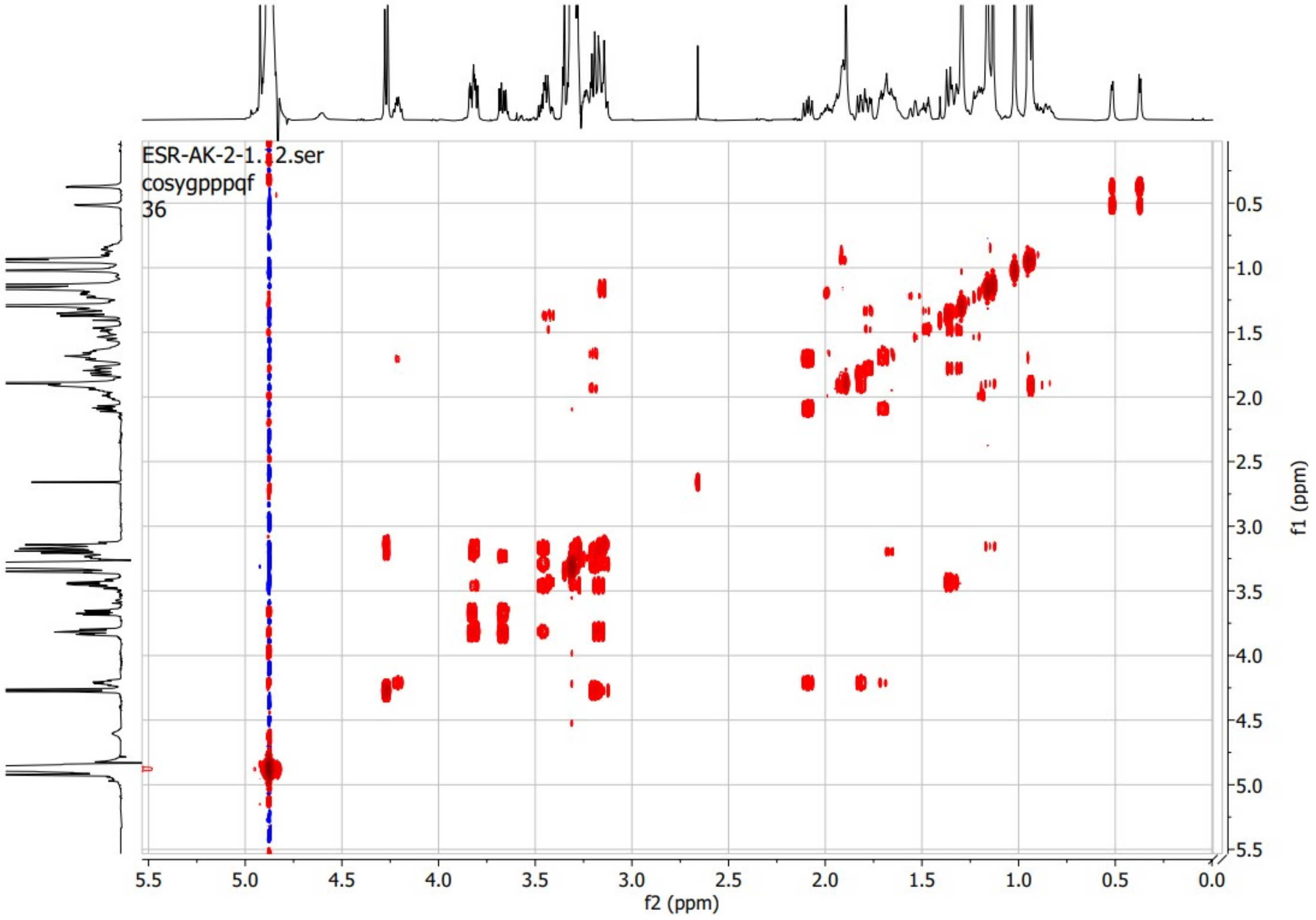

Figure S5: AN1 COSY spectra

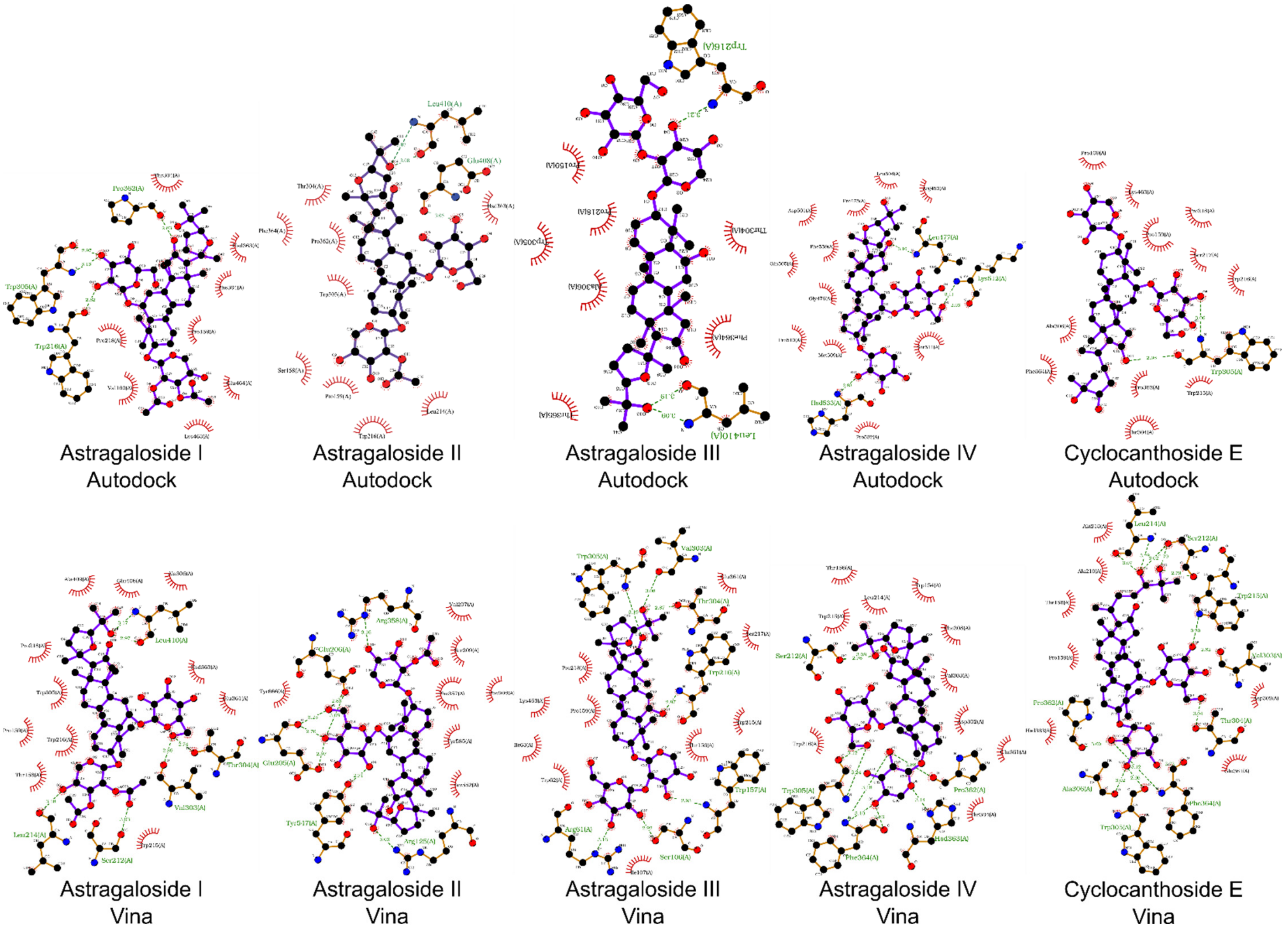

Figure S6: 2D interaction maps for the molecules astragaloside I-IV and cyclocanthoside E blind docked on chain-A of 1J2E.

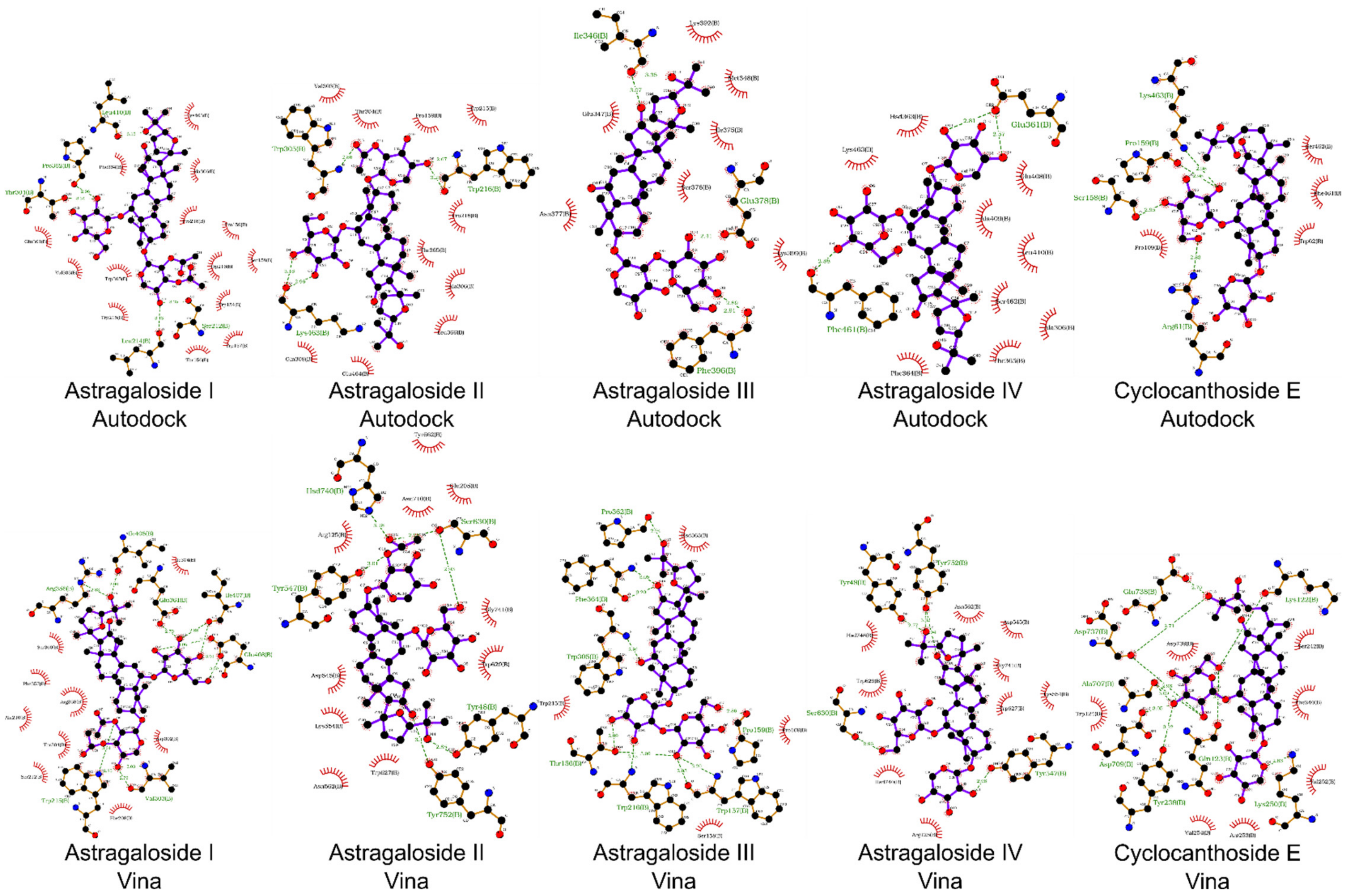

Figure S7: 2D interaction maps for the molecules astragaloside I-IV and cyclocanthoside E blind docked on chain-B of 1J2E.

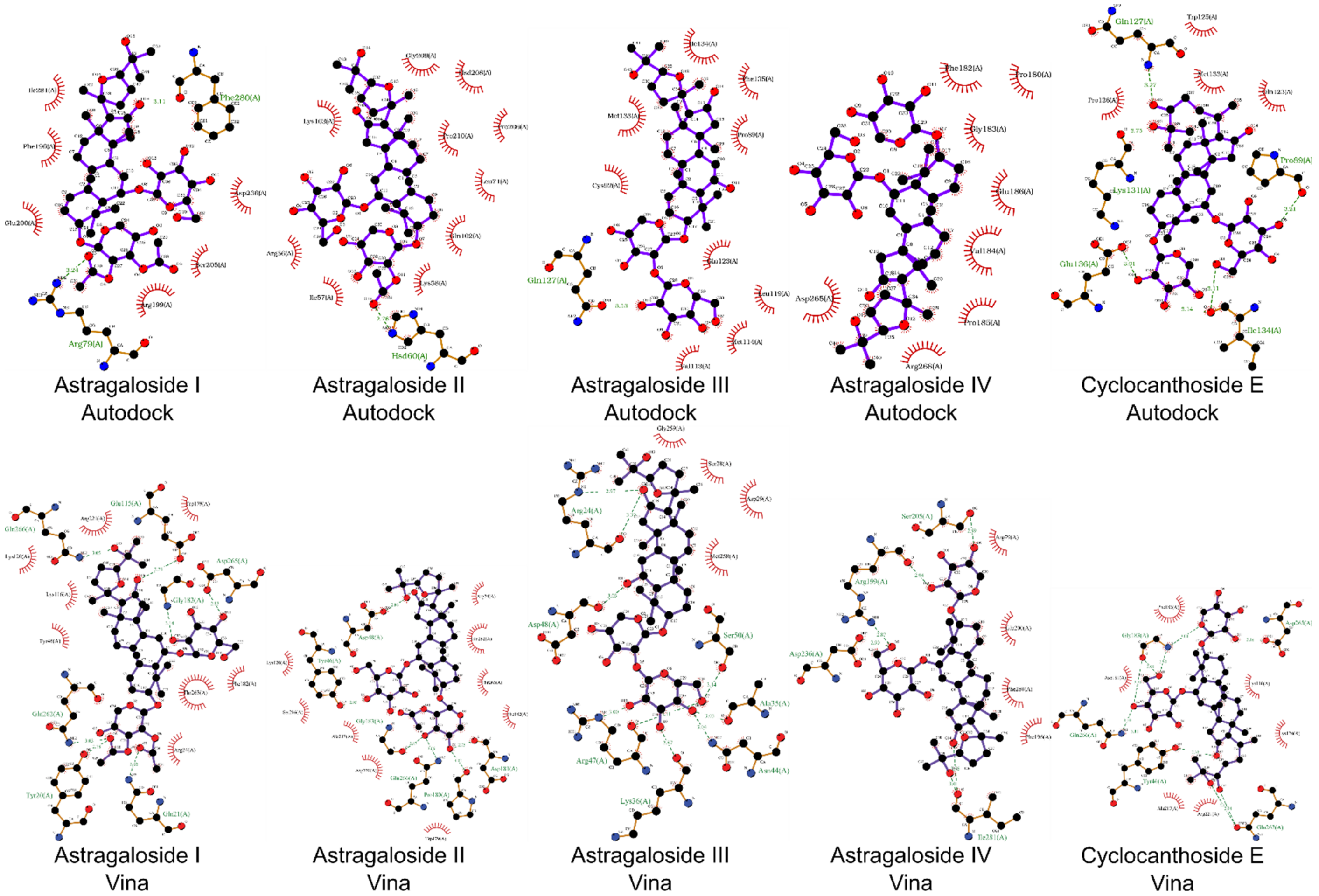

Figure S8: 2D interaction maps for the molecules astragaloside I-IV and cyclocanthoside E blind docked on 1NNY.

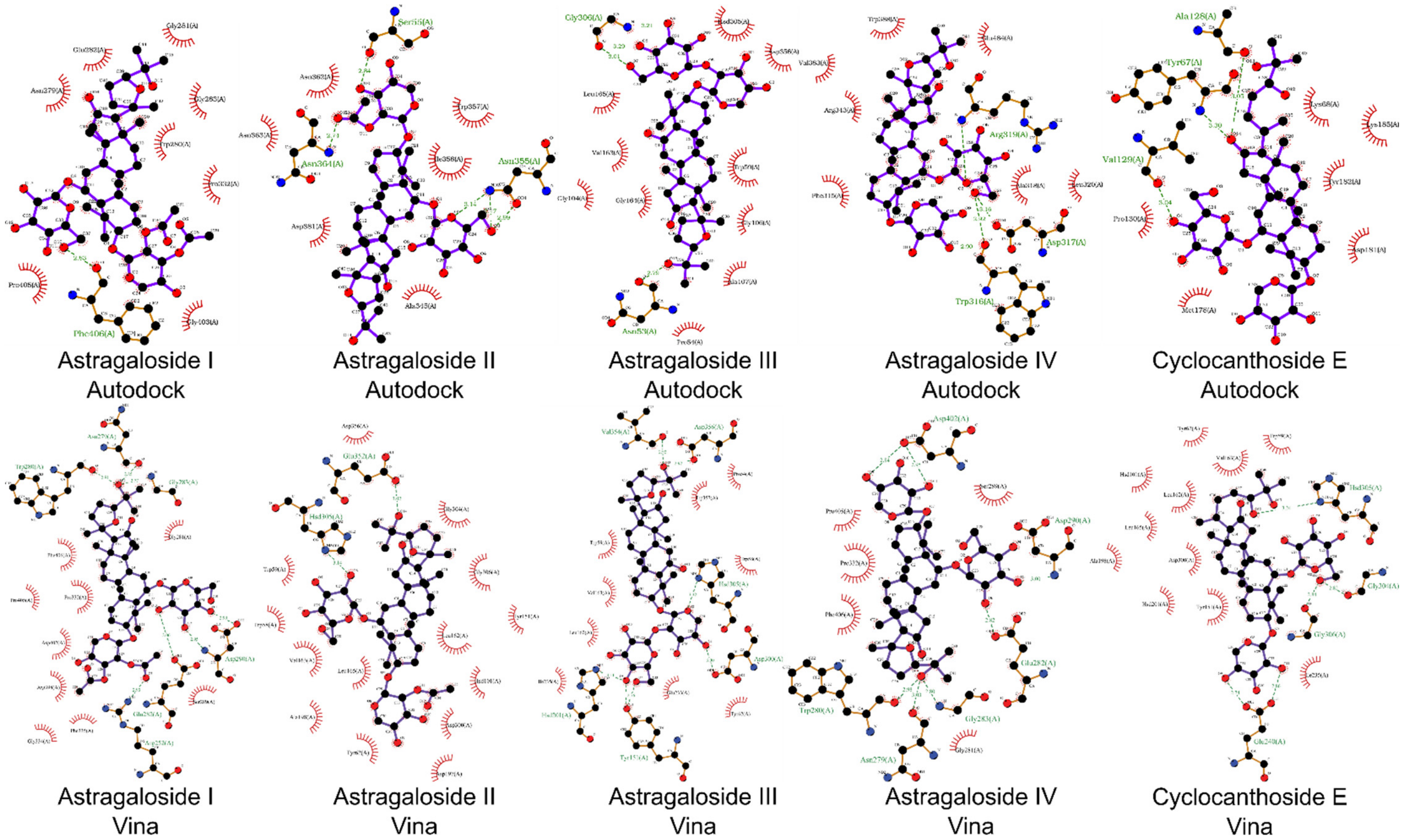

Figure S9: 2D interaction maps for the molecules astragaloside I-IV and cyclocanthoside E blind docked on 1OSE.

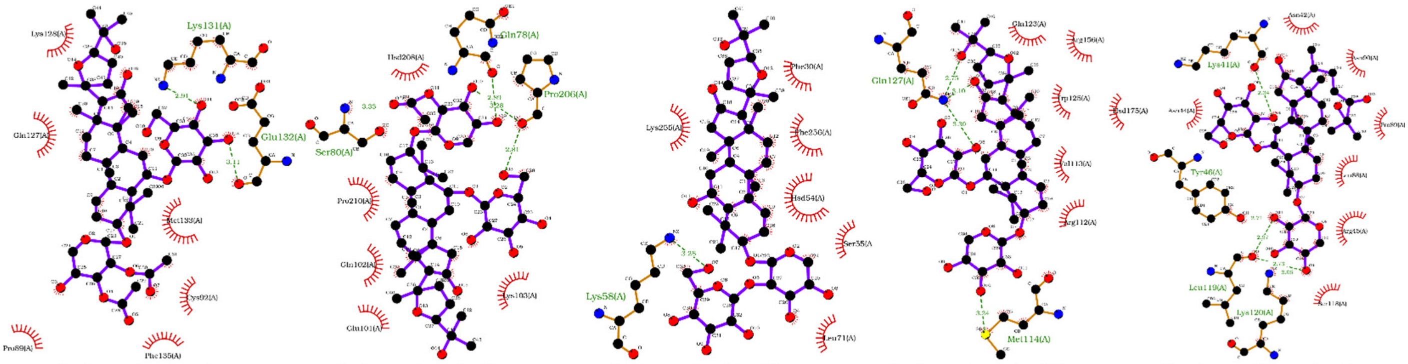

Astragaloside I Autodock | Astragaloside II Autodock | Astragaloside III Autodock | Astragaloside IV Autodock | Cyclocanthoside E Autodock

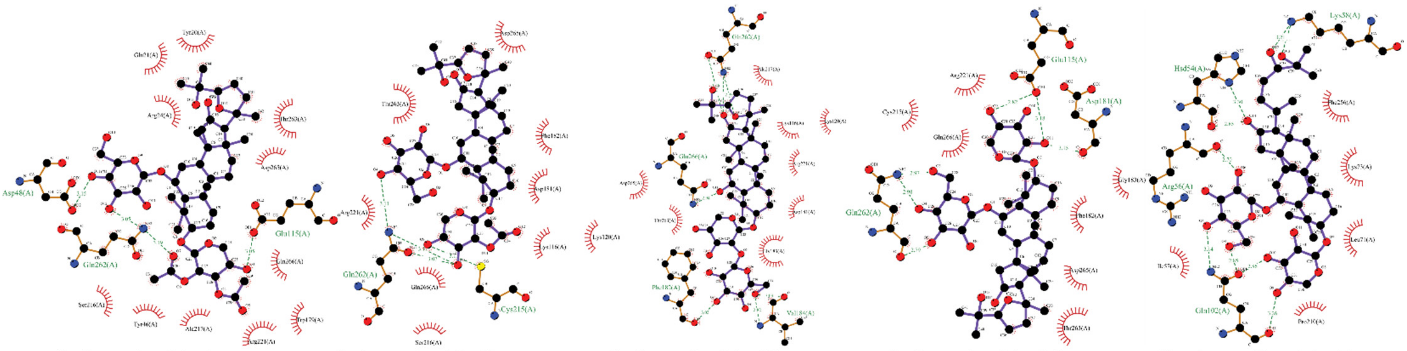

Astragaloside I Vina | Astragaloside II Vina | Astragaloside III Vina | Astragaloside IV Vina | Cyclocanthoside E Vina

Figure S10: 2D interaction maps for the molecules astragaloside I-IV and cyclocanthoside E blind docked on 1T49.

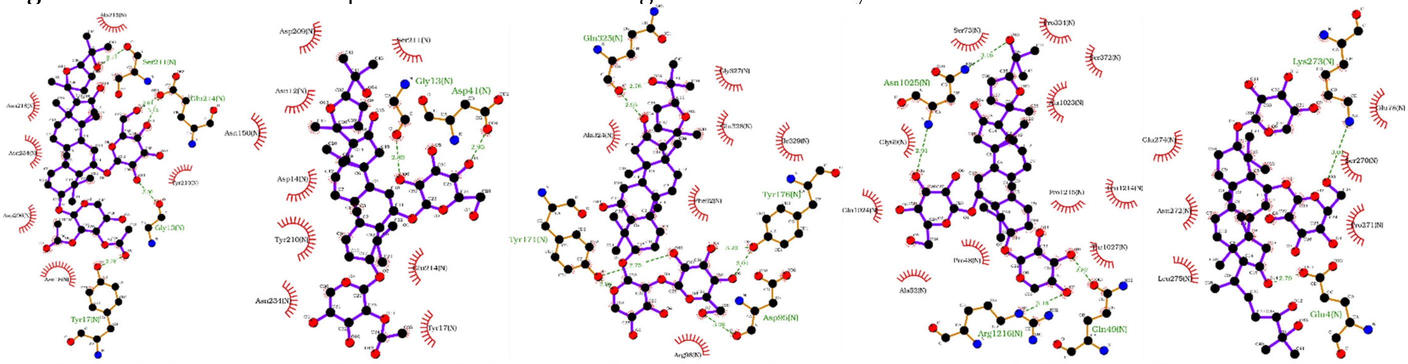

Astragaloside I Autodock | Astragaloside II Autodock | Astragaloside III Autodock | Astragaloside IV Autodock | Cyclocanthoside E Autodock

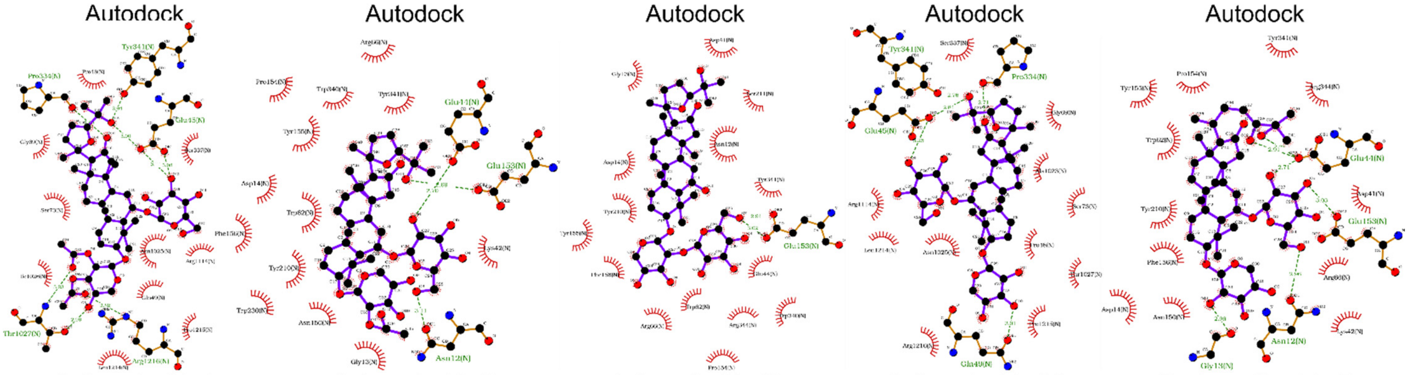

Astragaloside I Vina | Astragaloside II Vina | Astragaloside III Vina | Astragaloside IV Vina | Cyclocanthoside E Vina

Figure S11: 2D interaction maps for the molecules astragaloside I-IV and cyclocanthoside E blind docked on chain-A of 3O3U.

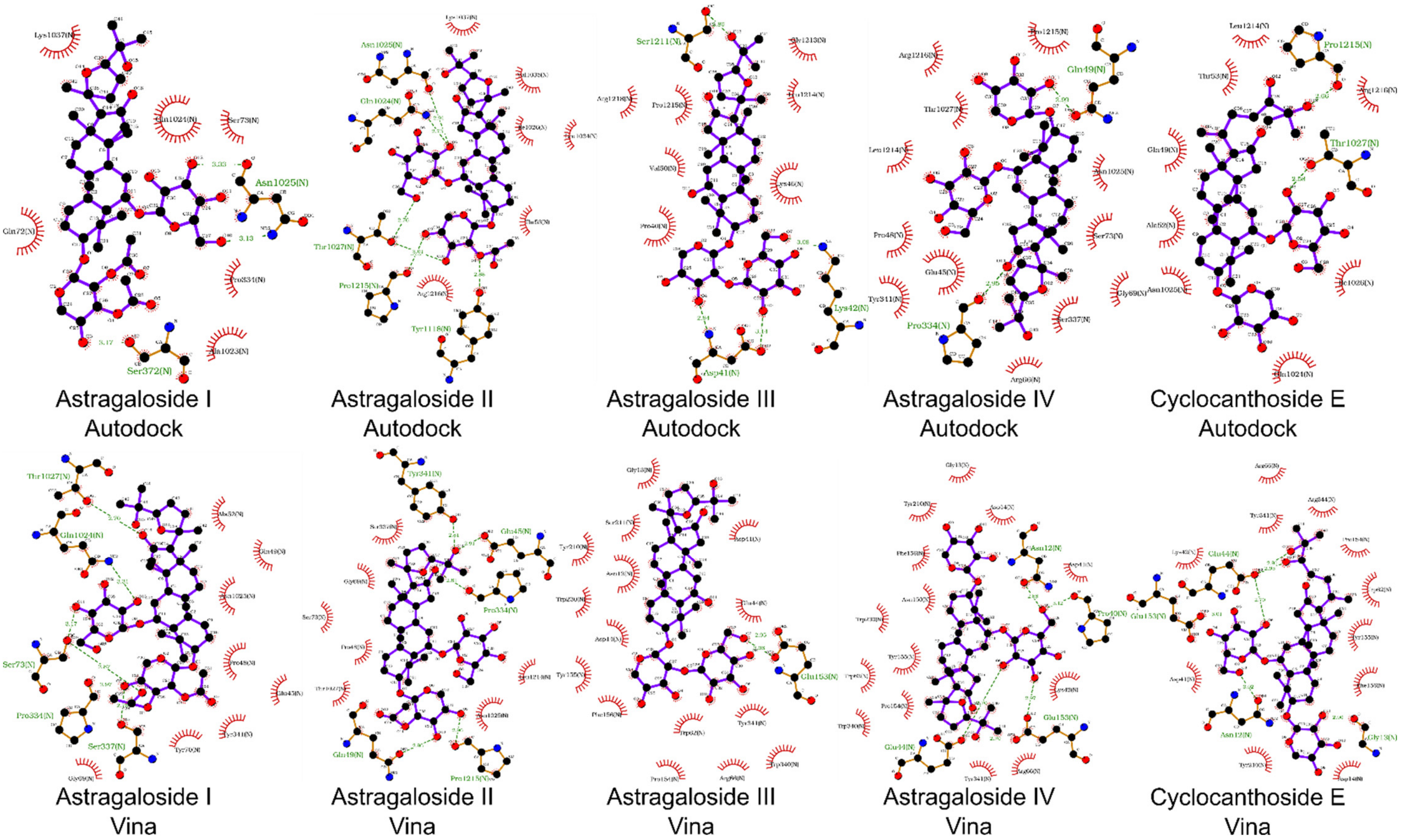

Figure S12: 2D interaction maps for the molecules astragaloside I-IV and cyclocanthoside E blind docked on chain-B of 3O3U.

Table S1. Percent inhibition values of the controls

	Standard					
	**1000µg/ml**	**500µg/ml**	**250µg/ml**	**125µg/ml**	**62.5µg/ml**	**31.25µg/ml**
α-amylase % Inhibition[#]	92.40 ± 0.07	88.89 ± 0.01	88.41 ± 0.01	82.56 ± 0.40	76.73 ± 0.07	-
PTP1B % Inhibition[##]	93.62 ± 0.07	90.75 ± 0.07	86.88 ± 0.35	84.53 ± 0.28	82.76 ± 0.01	73.55 ± 0.07
DPP4% Inhibition[§]	98.46 ± 0.07	93.21 ± 0.01	85.75 ± 0.28	79.53 ± 0.37	76.81 ± 0.04	73.15 ± 0.05
AGEs % Inhibition[§§]	93.82 ± 6.62	85.44 ± 6.88	64.39 ± 6.30	46.18 ± 13.82	26.72 ± 10.12	-

Acarbose. ## Ursolic Acid. § Vildagliptin. §§ Quercetin

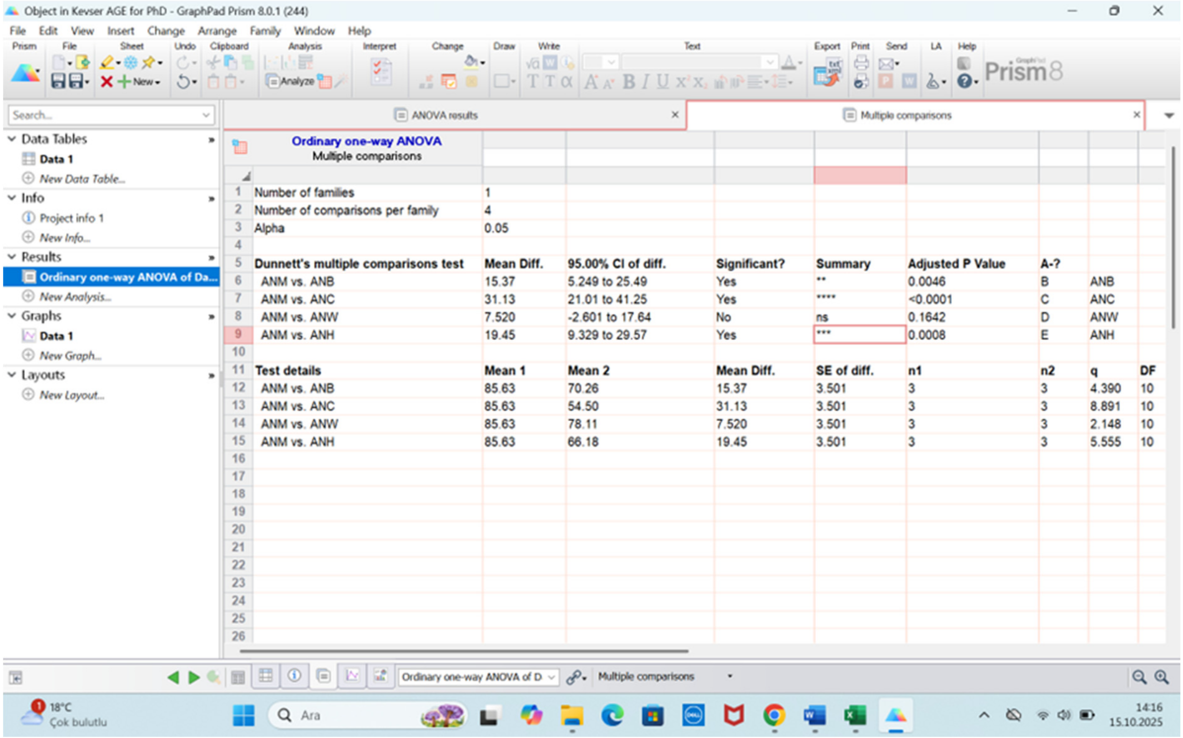

Supplementary Figure S13. Ordinary one-way ANOVA results for the AGE-formation inhibition assay of Astragalus noeanus extracts, analyzed using GraphPad Prism version 8.0.1.

Dunnett's multiple-comparison test was applied with ANM (methanolic extract) set as the reference group because it exhibited the highest mean inhibition (85.63 ± 3.50%). Pairwise comparisons revealed that ANB, ANC, and ANH showed significantly lower inhibition activities (adjusted P = 0.0046, < 0.0001, and 0.0008, respectively), whereas ANW displayed a statistically comparable inhibition level (adjusted P = 0.1642) and was therefore labeled as "ns" (non-significant).

This annotation indicates that although ANM showed the highest inhibition, the inhibitory capacity of ANW was statistically comparable within the experimental variation.

Data are presented as mean ± SD (n = 3) and analyzed by one-way ANOVA followed by Dunnett's post-hoc test (P < 0.05 considered significant).
